# Study of the preanalytical variables affecting the measurement of clinically relevant free-circulating microRNAs: focus on sample matrix, platelet depletion, and storage conditions

**DOI:** 10.11613/BM.2020.010703

**Published:** 2019-12-15

**Authors:** Martina Faraldi, Veronica Sansoni, Silvia Perego, Marta Gomarasca, Jakub Kortas, Ewa Ziemann, Giuseppe Banfi, Giovanni Lombardi

**Affiliations:** 1 Laboratory of Experimental Biochemistry & Molecular Biology, IRCCS Istituto ortopedico Galeazzi, Milano, Italia; 2 Department of Physical Culture, Gdańsk University of Physical Education & Sport, Gdańsk, Poland; 3 Department of Athletics, Strength and Conditioning, Poznan University of Physical Education, Poznan, Poland; 4 Università Vita-Salute San Raffaele, Milano, Italia

**Keywords:** circulating microRNA, biomarkers, plasma, preanalytical phase

## Abstract

**Introduction:**

Circulating microRNAs (miRNAs) are emerging as potential biomarkers. However, the lack of preanalytical and analytical standardization limits their use. The aim of this study was to determine the expression of different miRNAs in plasma according to different collection and storage conditions.

**Materials and methods:**

Venous blood from 10 volunteers was collected in tubes spray-coated with dipotassium salt of ethylendiaminetetraacetic acid, either with (plasma-preparation tube, PPT) or without (K2EDTA) gel separator. Platelet-poor plasma (PPP) was also obtained from K2EDTA plasma. After storage under different conditions, miRNA-enriched total RNA was isolated from plasma and reverse transcribed. A panel of 179 miRNAs was assayed by quantitative polymerase chain reaction and the results were analysed by GenEx software. Detectability and stability of miRNAs were determined.

**Results:**

The number of undetected miRNAs was: 18, 24, and 22 in PPT; 83, 43, and 20 in K2EDTA; and 76, 106, and 104 in PPP samples, for plasma immediately frozen at - 80°C and plasma stored for 24h at room temperature or 4°C, respectively. Circulating miRNA expression in PPT samples was not affected by storage delay or temperature, while the percentage of up- and down-regulated miRNA in K2EDTA and PPP samples ranged from 2%, and 1% to 7%, and 5%, respectively.

**Conclusions:**

Sample matrix, temperature and delay in storage strongly influence the expression level of plasma miRNAs. Our results indicate PPT tubes as the most suitable matrix to improve total miRNA detectability and stability, independently of temperature.

## Introduction

In order to be benchmarked, a novel biomarker should be: i) measurable in a specific clinical setting, ii) validated by multiple studies, iii) directly impacting on the medical decision (*1*). Although they do not yet satisfy all these criteria, microRNAs (miRNAs), and circulating miRNAs in particular, have gained a place among new-generation biomarkers. Circulating miRNAs can be measured in 12 types of body fluids (*e.g.*, plasma and serum where they are particularly abundant, blood, urine, cerebrospinal fluid, saliva) and 61 different types of tissues (*2*, *3*). Nonetheless, their validation and impact on medical decision criteria are still lacking. Validation is hindered by the lack of analytical (*i.e.*, low inter-platform concordance) and postanalytical (*i.e.*, normalization strategy) standardization (*4*, *5*). Their impact on medical decision is weakened by uncertainty about the meaning of the changes in their expression: being fine-tuners of gene expression, miRNA expression is highly variable (*6*).

Despite these limitations, the abnormal expression of a single miRNA or miRNA families has been more or less specifically associated with disorders such as cancer, cardiovascular diseases, inflammatory and autoimmune diseases (*7*). Indeed, the diagnostic potential of the whole miRNAs expression profile (“miRNome”) could be even greater. Hence, given their key biological role, it is desirable to increase their implementation in routine diagnostics. To this end, standardization of preanalytical and postanalytical phases are needed (*6*, *8*). Furthermore, current knowledge is still poor about the preanalytical warnings concerning samples intended for miRNAs analysis.

Circulating miRNAs can be passively released from cells because of physiological renewal or cell death, constituting free-circulating miRNAs associated with carrier proteins such as argonaute (Ago)-2 and nucleophosmin (NPM)-1, or they can be actively secreted encapsulated within microvesicles/exosomes targeting other cells/tissues (*9*). Their concentration in plasma, as estimated by quantitative real-time polymerase chain reaction (qPCR), is physiologically highly variable and ranges between 1 and 308 μg/L (*10*, *11*). Therefore, any variable other than the intrinsic biological variables would impact on the outcome of miRNAs expression (*12*).

Among the still unsolved questions about the preanalytical management of samples for miRNA analysis, the choice of sample matrix poses multiple challenges. Although this has never been considered as a real issue in practical terms, it does have implications for the final analytical output (*6*). Previous studies have demonstrated the stability of miRNAs in serum and plasma, with a higher variability for the former than the latter, as also recently confirmed by Basso *et al.* (*13*-*16*). For plasma, the choice of the anticoagulant is pivotal. Because heparin and citrate interfere with the enzyme activities in PCR, they are not recommended for qPCR-based miRNAs quantitation (*2*). However, heparinase treatment improves the results in heparin-anticoagulated plasma, although Basso *et al.* reported a severe interference by heparin (*15*, *16*). In addition, ethylendiaminetetraacetate (EDTA) potassium salts (K2 and K3), which can be removed from the PCR mastermix, are regarded as the best anticoagulants for assaying circulating miRNAs (*17*). Sodium fluoride/potassium oxalate (NaF/KOx), an alternative to EDTA, increases miRNA detection (*15*).

Against this background, we investigated the preanalytical variables, including sample matrix, time- and temperature-dependent stability, in the measurement of a wide panel of circulating miRNAs in EDTA-anticoagulated plasma. In addition, we checked for possible contamination from platelet-derived miRNAs. To this end we assayed: i) two different EDTA-anticoagulated sample matrices either with or without gel separator (plasma preparation tubes (PPT) and K2EDTA tubes, respectively); ii) two different centrifugation settings for plasma separation (single standard and single standard plus platelet-depleting centrifugation); iii) three different plasma storage conditions (immediate freezing at - 80°C, and storage for 24h at either room temperature or 4°C).

## Materials and methods

### Study design and subjects

This observational study was designed to determine the stability and quantification of circulating miRNAs in plasma subjected to different preanalytical variables, such as sample collection and storage conditions.

In this study, ten adult male volunteers (aged 25 – 40 years) were recruited among the personnel of the IRCCS Istituto Ortopedico Galeazzi, between November 2016 and January 2017. Eligibility criteria were: global health status with no acute or chronic disease and no chronic or current ongoing medical treatment, normal body-mass index (20 kg/m^2^ ≤ BMI < 25 kg/m^2^), and non-smoking. Subjects were asked to refrain from alcohol intake, physical activity, pro-inflammatory activities (*e.g.*, shaving and waxing) in the 48h preceding blood drawing, and to keep a regular diet.

Venous blood was collected in K2EDTA and PPT (BD Vacutainer, Becton Dickinson, Milan, Italy). Samples were centrifuged at standard condition of 1300xg for 10 min at room temperature (RT) of 22°C. The plasma was collected and an aliquot from the K2EDTA tubes was further centrifuged at 2500xg for 15 min at RT to eliminate platelet residues and to obtain the platelet-poor plasma (PPP) (*18*). Plasma aliquots from K2EDTA, PPP, and PPT were frozen at - 80°C either immediately or after storage for 24 h at either RT or 4°C. The protocol was approved by the ethical committee (Comitato Etico Ospedale San Raffaele, Milano, on 4 February 2016) and registered with ClinicalTrials.gov (registration number NCT03386981, 4th January 2018). Written, informed consent to participate was obtained from all subjects after being informed about procedures, risks, and benefits associated with the research, as required by the Helsinki Declaration.

### Methods

Total plasma RNA enriched in miRNAs was isolated with the miRCURY RNA Isolation Kit (Exiqon A/S, Vedbaek, Denmark) and reverse transcription was carried out using miRCURY LNA™ Universal reverse transcription miRNA PCR (Exiqon A/S, Vedbaek, Denmark). The spike-in controls UniSp2, UniSp4, UniSp5, and UniSp6 and cel-39 (Exiqon A/S, Vedbaek, Denmark), added to each sample at recommended concentration, were used to check the efficiency of RNA extraction and reverse transcription, respectively. Real-time qPCR (StepOne Plus, Applied Biosystems, Foster City, USA) was performed with ExiLENT SYBR Green Master Mix (Exiqon A/S, Vedbaek, Denmark) and a primer set of the 179 miRNAs most highly represented in the circulation (serum/plasma miRCURY LNA miRNA focus panel, Exiqon A/S, Vedbaek, Denmark). Three inter-plate calibrators (IPC), with specific primer pairs and DNA template, were provided with the panel kit. The PCR was conducted over 40 cycles (10 sec at 95°C, 60 sec at 60°C) preceded by 10-min activation of the polymerase at 95°C and followed the melting curves. Each sample was analysed in triplicate.

Reverse transcription-qPCR data analysis was conducted as previously described (*8*). Briefly, the threshold cycle (C_T_) values from qPCR were analysed with GenEx software v6 (Exiqon A/S, Vedbaek, Denmark). Threshold cycle values from the plate runs of different experiments were adjusted based on IPC C_T_ values. Only those miRNAs with a C_T_ < 37 were further considered in the analysis. The 2^−ΔΔCT^ method was applied to calculate the relative expression of each miRNA and the global mean (*i.e.*, the geometrical mean of the whole dataset C_T_ values) was used to normalize the data, as this strategy is defined as being highly reliable in this specific experimental setting according to Faraldi *et al*. (*8*). The C_T_ difference between hsa-miR-23a and hsa-miR-451 was used to check for micro-haemolysis, which can eventually affect the analysis (*19*). None of the samples exceeded the cut-off value of 7.

Comparisons of detected and undetected miRNAs in the different analysed conditions were performed to define miRNA detectability. Fold change (FC, the ratio between the normalized outputs from an “experimental” condition and “reference” condition) analyses were performed to determine the differences in circulating miRNA expression in all the different conditions. More specifically, we focused on the measurement of clinically relevant miRNAs: miRNAs associated with bone fracture risk; skeletal muscle modulated, both in tissue and circulation, during physical activity; and miRNAs associated with cancer diagnosis (with a reported area under curve (AUC) > 0.70) (*20*-*23*). Results are shown only for the miRNAs that were significantly ≥ 5-fold either up- or down-regulated, in at least one condition.

### Statistical analysis

Detected and undetected miRNAs were compared within- and between-group considering the sample collection or the storage condition, using Pearson’s Chi-Square test with Yates’ continuity correction. Additionally, both Pearson’s Chi-Square test with Yates’ continuity correction and McNemar’s Chi-square test with continuity correction, for paired data, were performed comparing two conditions at a time. The non parametric Kruskal-Wallis test with Dunn’s multiple comparison test was used for within- and between-group comparison of the expression of each miRNA. Differences were considered statistically significant if P-values < 0.05. Statistical analysis was performed with Statistical 13.1 software (Statsoft, Tulsa, USA).

## Results

### Effect of sample matrix on samples immediately frozen at - 80°C

The number of undetected miRNAs in plasma collected in K2EDTA tubes was significantly higher compared to plasma in PPT tubes immediately frozen at - 80°C (as shown in Table 1).

**Table 1:** Detected and undetected miRNAs of samples collected in K2EDTA and PPT, immediately frozen at - 80°C

**Storage temperature**	**miRNAs**	**K2EDTA, N (%)** **N = 179**	**PPT, N (%)** **N = 179**	**χ^2^ Test**	**P**
- 80°C	detected	96 (54)	161 (90)	56.49	< 0.001
	undetected	83 (46)	18 (10)		
Pearson’s Chi-Square test with Yates’ continuity correction and relative P-value are reported. P < 0.05 was considered statistically significant. K2EDTA - dipotassium ethylendiaminetetraacetate tubes. PPT – plasma preparation tubes.					

Because 15 miRNAs (hsa-miR-100-5p, hsa-miR-133a, hsa-miR136-3p, hsa-miR136-5p, hsa-miR-141-3p, hsa-miR-20b-5p, hsa-miR-200a-5p, hsa-miR-208-3p, hsa-miR22-5p, hsa-miR-33a-5p, hsa-miR-335-5p, hsa-miR-362, hsa-miR-365a, hsa-miR497-5p, and hsa-miR-7-1-3p) were undetectable in both conditions, all the following analyses were performed on 164 miRNAs. Two miRNAs (1% of the 164 miRNAs) were either up- or down-regulated in PPT compared to K2EDTA samples (Table 2).

**Table 2:** Between-group comparison of circulating miRNA expression

	**Storage condition**											
**miRNAs,**	**immediately frozen at - 80°C**				**24h RT**				**24h 4°C**			
**fold change (P)**	**PPT *vs* K2EDTA**	**K2EDTA *vs* PPP**	**PPT *vs* PPP**	**P**	**PPP *vs* K2EDTA**	**PPT *vs* K2EDTA**	**PPT *vs* PPP**	**P**	**PPP *vs* K2EDTA**	**PPT *vs* K2EDTA**	**PPT *vs* PPP**	**P**
hsa-miR-1	12.84	0.02	0.29	0.092	0.02 (0.027)	0.12 (0.470)	0.16 (0.699)	0.032	0.02 (0.027)	0.20 (0.470)	0.11 (0.699)	0.032
hsa-miR-106a-5p	1.36	1.78	2.41	0.051	0.60 (0.539)	3.62 (0.539)	0.17 (0.022)	0.027	0.73	0.58	4.41	0.079
hsa-miR-10b-5p	4.36	1.00	4.36	1.000	323.51 (0.022)	23.92 (0.539)	13.52 (0.539)	0.027	203.30 (0.022)	35.11 (0.539)	15.03 (0.539)	0.027
hsa-miR-122-5p	0.80	0.37	0.30	0.061	3.31 (0.539)	0.34 (0.539)	9.71 (0.022)	0.027	2.22 (0.539)	0.33 (0.539)	0.023 (0.022)	0.027
hsa-miR-125b-5p	0.22 (0.539)	169.01 (0.022)	36.73 (0.539)	0.027	1.00	21.01	0.05	0.063	0.02	0.03	0.52	0.064
hsa-miR-1260a	43.13	0.94	40.37	0.058	0.01 (0.022)	0.42 (0.539)	0.03 (0.539)	0.027	0.01 (0.022)	0.79 (0.539)	0.58 (0.539)	0.027
hsa-miR-128-3p	1.00	1.12	1.12	0.869	1.01	53.70	0.02	0.064	0.02 (0.028)	0.71 (0.539)	1.17 (0.539)	0.027
hsa-miR-140-5p	41.24	1.00	41.24	0.063	1.00	32.90	0.03	0.063	1.10 (0.699)	41.32 (0.027)	36.22 (0.470)	0.032
hsa-miR-143-3p	34.31 (0.539)	0.01 (0.022)	0.22 (0.539)	0.027	1.38	28.73	0.05	0.055	0.01	0.98	0.25	0.067
hsa-miR-144-3p	0.17 (0.034)	1.74 (0.890)	0.29 (0.408)	0.039	6.30	0.51	12.43	0.148	2.31	1.63	0.19	0.491
hsa-miR-144-5p	53.84	1.04	56.03	0.064	0.01 (0.034)	0.45 (0.890)	0.02 (0.408)	0.039	1.62 (0.539)	0.27 (0.539)	76.36 (0.022)	0.027
hsa-miR-146b-5p	32.94 (0.539)	0.01 (0.022)	0.26 (0.539)	0.027	0.01 (0.022)	0.49 (0.539)	0.02 (0.539)	0.027	0.01	0.83	0.63	0.061
hsa-miR-152-3p	0.47 (0.539)	106.31 (0.022)	50.45 (0.539)	0.027	1.81	1.03	1.75	0.067	0.02 (0.408)	2.70 (0.890)	0.01 (0.034)	0.039
hsa-miR-155-5p	56.78 (0.408)	0.01 (0.034)	0.41 (0.890)	0.039	0.01	0.61	0.02	0.067	1.10	67.05	62.57	0.064
hsa-miR-15a-5p	1.97 (0.539)	0.20 (0.022)	0.39 (0.539)	0.027	2.40	0.81	2.95	0.061	1.21	0.43	0.41	0.061
hsa-miR-15b-3p	63.95	1.00	63.95	0.063	0.02 (0.034)	0.55 (0.890)	0.03 (0.408)	0.039	1.76 (0.539)	0.54 (0.539)	56.45 (0.022)	0.027
hsa-miR-16-2-3p	45.83 (0.539)	0.01 (0.022)	0.26 (0.539)	0.027	0.01	0.01	1.00	0.063	0.01	0.02	0.01	0.058
hsa-miR-186-5p	1.21	0.83	1.00	0.965	0.01 (0.022)	0.35 (0.539)	0.03 (0.539)	0.027	0.58	1.21	21.01	0.634
hsa-miR-191-5p	1.94 (0.890)	3.57 (0.408)	6.94 (0.034)	0.039	0.16	1.15	0.14	0.051	0.34 (0.890)	1.67 (0.408)	2.42 (0.034)	0.039
hsa-miR-194-5p	28.90 (0.539)	0.01 (0.022)	0.16 (0.539)	0.027	5.78 (0.539)	0.39 (0.539)	14.87 (0.022)	0.027	2.63 (0.539)	0.53 (0.539)	0.18 (0.022)	0.027
hsa-miR-195-5p	46.57	1.00	46.57	0.063	0.01 (0.022)	0.54 (0.539)	0.03 (0.539)	0.027	0.01 (0.303)	2.48 (0.990)	0.34 (0.051)	0.051
hsa-miR-199a-5p	0.46 (0.539)	60.84 (0.022)	28.07 (0.539)	0.027	0.87	48.86	0.02	0.064	0.87	49.74	48.86	0.064
	**Storage condition**											
**miRNAs,**	**immediately frozen at - 80°C**				**24h RT**				**24h 4°C**			
**fold change (P)**	**PPT *vs* K2EDTA**	**K2EDTA *vs* PPP**	**PPT *vs* PPP**	**P**	**PPP *vs* K2EDTA**	**PPT *vs* K2EDTA**	**PPT *vs* PPP**	**P**	**PPP *vs* K2EDTA**	**PPT *vs* K2EDTA**	**PPT *vs* PPP**	**P**
hsa-miR-210-3p	33.68 (0.539)	0.005 (0.022)	0.16 (0.539)	0.027	1.00	1.17	0.85	0.723	0.02	0.02	0.02	0.058
hsa-miR-2110	42.84	0.01	0.52	0.082	0.01 (0.034)	0.40 (0.890)	0.03 (0.408)	0.039	0.01 (0.022)	0.48 (0.539)	0.44 (0.539)	0.027
hsa-miR-215-5p	0.36 (0.303)	0.87 (0.990)	0.31 (0.051)	0.051	1.16	33.39	0.03	0.064	3.25 (0.539)	0.66 (0.539)	93.70 (0.022)	0.027
hsa-miR-21-5p	1.61	1.08	1.75	0.067	1.20	1.17	1.02	0.202	0.20 (0.539)	1.64 (0.539)	0.19 (0.022)	0.027
hsa-miR-223-3p	1.53 (0.539)	1.61 (0.539)	2.48 (0.022)	0.027	0.07	1.55 (0.539)	0.04 (0.022)	0.027 (0.539)	0.15	0.95	3.42	0.067
hsa-miR-24-3p	1.54 (0.539)	3.51 (0.539)	5.39 (0.022)	0.027	0.20 (0.303)	1.12 (0.990)	0.18 (0.051)	0.051	0.66 (0.539)	2.45 (0.539)	3.67 (0.022)	0.027
hsa-miR-29c-3p	55.34 (0.539)	0.004 (0.022)	0.21 (0.539)	0.027	0.01	0.01	1.00	0.063	0.01	0.75	0.01	0.061
hsa-miR-301a-3p	0.36 (0.539)	170.97 (0.022)	61.12 (0.539)	0.027	0.01 (0.034)	0.71 (0.890)	0.02 (0.408)	0.039	0.02 (0.539)	2.54 (0.539)	1.13 (0.022)	0.027
hsa-miR-30a-5p	34.37	1.07	36.71	0.058	358.44 (0.022)	40.39 (0.539)	8.87 (0.539)	0.027	4.54	1.14	0.51	0.061
hsa-miR-30e-3p	38.32 (0.539)	38.32 (0.022)	0.33 (0.539)	0.027	0.01 (0.034)	0.46 (0.890)	0.02 (0.408)	0.039	0.02	1.75	0.72	0.061
hsa-miR-32-5p	0.02 (0.539)	0.47 (0.539)	0.01 (0.022)	0.027	0.02 (0.022)	0.53 (0.539)	0.04 (0.539)	0.027	1.10	6.99	28.49	0.107
hsa-miR-331-3p	0.01 (0.034)	1.64 (0.890)	0.01 (0.408)	0.039	0.02	0.95	0.02	0.061	0.03	1.63	1.76	0.061
hsa-miR-335-3p	50.45	0.86	43.19	0.058	0.01 (0.022)	0.32 (0.539)	0.03 (0.539)	0.027	0.02	1.36	0.87	0.067
hsa-miR-339-5p	0.58 (0.890)	119.87 (0.034)	69.93 (0.408)	0.039	0.01	1.01	0.01	0.067	0.02	1.10	2.22	0.067
hsa-miR-363-3p	0.45 (0.539)	138.59 (0.022)	63.02 (0.539)	0.027	4.91	0.71	6.96	0.061	1.44	0.64	0.21	0.252
hsa-miR-374a-5p	40.32 (0.470)	1.29 (0.699)	52.14 (0.027)	0.032	0.01 (0.051)	0.64	0.02	0.051	0.02	1.22	0.69	0.067
hsa-miR-374b-5p	1.22	49.60	60.46	0.061	0.03 (0.539)	2.06 (0.539)	0.01 (0.022)	0.027	0.02	0.96	1.45	0.067
hsa-miR-375	0.22 (0.539)	195.35 (0.022)	42.75 (0.539)	0.027	0.01 (0.022)	0.22 (0.539)	0.03 (0.539)	0.027	11.12 (0.539)	0.11 (0.539)	427.55 (0.028)	0.027
hsa-miR-378a-3p	0.14 (0.539)	0.51 (0.539)	0.07 (0.022)	0.027	4.73 (0.539)	0.39 (0.539)	12.24 (0.022)	0.027	2.20	0.57	0.18	0.079
hsa-miR-409-3p	67.32 (0.027)	0.89 (0.699)	59.60 (0.470)	0.032	0.01	0.43	0.01	0.061	0.02	1.42	1.33	0.067
hsa-miR-421	31.58	1.00	31.58	0.063	0.01 (0.034)	0.48 (0.890)	0.03 (0.408)	0.039	0.02 (0.022)	0.64 (0.539)	0.63 (0.539)	0.027
hsa-miR-424-5p	0.01	126.48	1.00	0.063	291.39 (0.022)	26.76 (0.539)	10.89 (0.539)	0.027	0.02	0.02	0.00	0.063
	**Storage condition**											
**miRNAs,**	**immediately frozen at - 80°C**				**24h RT**				**24h 4°C**			
**fold change (P)**	**PPT *vs* K2EDTA**	**K2EDTA *vs* PPP**	**PPT *vs* PPP**	**P**	**PPP *vs* K2EDTA**	**PPT *vs* K2EDTA**	**PPT *vs* PPP**	**P**	**PPP *vs* K2EDTA**	**PPT *vs* K2EDTA**	**PPT *vs* PPP**	**P**
hsa-miR-425-5p	91.97 (0.408)	0.01 (0.034)	0.50 (0.890)	0.039	0.01	0.62	0.01	0.067	1.28	0.89	124.48	0.561
hsa-miR-454-3p	49.88 (0.027)	0.76 (0.699)	37.92 (0.470)	0.032	0.01 (0.022)	0.50 (0.539)	0.03 (0.539)	0.027	0.02	0.63	0.88	0.063
hsa-miR-483-5p	39.02 (0.539)	0.04 (0.022)	0.14 (0.539)	0.027	2.29 (0.408)	0.27 (0.890)	8.59 (0.034)	0.039	0.01	0.96	0.00	0.061
hsa-miR-486-5p	1.59 (0.539)	0.23 (0.022)	0.37 (0.539)	0.027	1.79 (0.539)	0.21 (0.539)	8.35 (0.022)	0.027	3.73 (0.408)	0.81 (0.890)	0.45 (0.034)	0.039
hsa-miR-501-3p	1.28	0.78	1.00	0.723	0.01	0.01	0.84	0.064	0.02 (0.022)	0.40 (0.539)	0.02 (0.539)	0.027
hsa-miR-505-3p	38.61	1.00	38.61	0.063	0.01 (0.022)	0.21 (0.539)	0.02 (0.539)	0.027	0.01	0.01	0.38	0.055
hsa-miR-629-5p	0.20	0.72	0.15	0.55	5.01 (0.539)	0.23 (0.539)	21.97 (0.022)	0.027	0.02	0.45	0.00	0.055
hsa-miR-660-5p	1.00	1.00	1.00	1.000	3.45 (0.539)	0.02 (0.539)	164.86 (0.022)	0.027	0.02 (0.022)	0.54 (0.539)	0.00 (0.539)	0.027
hsa-miR-7-5p	13.02	0.02	0.29	0.092	1.00	6.20	0.16	0.106	0.02 (0.027)	0.15 (0.470)	0.15 (0.699)	0.032
hsa-miR-766-3p	0.52 (0.539)	63.76 (0.022)	33.10 (0.539)	0.027	0.01 (0.034)	0.78 (0.890)	0.02 (0.408)	0.039	0.02 (0.539)	3.35 (0.539)	1.45 (0.022)	0.027
hsa-miR-877-5p	37.53 (0.539)	0.012 (0.022)	0.45 (0.539)	0.027	0.74	33.42	0.02	0.063	0.01 (0.022)	0.56 (0.539)	0.38 (0.539)	0.027
hsa-miR-885-5p	7.21 (0.699)	0.01 (0.027)	0.06 (0.470)	0.032	1.00	6.18	0.16	0.106	0.02	0.59	0.14	0.129
hsa-miR-93-3p	78.27	0.01	0.77	0.061	0.01 (0.034)	0.52 (0.890)	0.03 (0.408)	0.039	2.01	0.77	73.21	0.061
hsa-miR-99a-5p	1.15	0.87	1.00	0.723	0.01 (0.022)	0.48 (0.539)	0.02 (0.539)	0.027	0.02 (0.051)	0.79 (0.990)	1.06 (0.303)	0.051
hsa-miR-99b-5p	1.79 (0.890)	50.77 (0.408)	90.83 (0.034)	0.039	0.01	1.37	0.01	0.067	0.03 (0.408)	1.97 (0.890)	3.22 (0.034)	0.039
Expressions for each miRNAs from K2EDTA, PPP and PPT samples and each storage condition were compared through Kruskal-Wallis test. P < 0.05 was considered statistically significant. Dunn’s multiple comparison test was performed for comparison between two storage conditions, only for significant Kruskal-Wallis test. Differences in miRNA expression between two conditions is reported as the ratio of miRNA expression of an “experimental” condition and a “reference” condition (fold change). Results are reported only for miRNAs with a statistically significant ≥ ± 5-fold change, in at least one comparison. K2EDTA - dipotassium ethylendiaminetetraacetate tubes. PPT – plasma preparation tubes. PPP- platelet-poor plasma.												

### Effect of platelet depletion on samples immediately frozen at - 80°C

Platelet depletion was highlighted by the lower plasma expression of hsa-miR-126-3p, hsa-miR-223-3p, and hsa-miR-27b-3p, three of the most highly expressed miRNAs in platelets, in PPP samples compared to K2EDTA and PPT (Table 2, Supplementary Table 1) (*24*, *25*).

Considerable differences were observed in the number of undetected miRNAs in K2EDTA, PPT, and PPP samples when immediately frozen at - 80°C (Table 3): specifically, the stepwise centrifugation significantly increased the frequency of undetected miRNAs compared to PPT samples (Supplementary Table 2 and 4). The 15 undetected miRNAs in the K2EDTA and PPT samples were undetected also in the PPP samples. The FC analyses showed that the percentage of up- and down-regulated miRNAs in K2EDTA as compared to PPP samples were 5% and 7%, respectively (Table 2), while in the PPT as compared to the PPP samples were 2% and 1%, respectively (Table 2).

**Table 3:** Between-group comparison of detected and undetected miRNAs

**Storage condition**	**miRNAs**	**K2EDTA, N (%)** **N = 179**	**PPP, N (%)** **N = 179**	**PPT, N (%)** **N = 179**	**χ^2^ Test**	**P**
- 80°C	detected	96 (54)	103 (58)	161 (90)	64.37	< 0.001
	undetected	83 (46)	76 (42)	18 (10)		
24h RT	detected	136 (76)	73 (41)	155 (87)	94.26	< 0.001
	undetected	43 (24)	106 (59)	24 (13)		
24h 4°C	detected	159 (89)	75 (42)	157 (88)	129.66	< 0.001
	undetected	20 (11)	104 (58)	22 (12)		
Pearson’s Chi-Square test with Yates’ continuity correction was performed for detected and undetected miRNAs from samples immediately stored at - 80°C, stored for 24h at RT, and stored for 24h at 4°C among the different collection conditions (K2EDTA, PPP and PPT). P < 0.05 was considered statistically significant. RT – room temperature. K2EDTA - dipotassium ethylendiaminetetraacetate tubes. PPT – plasma preparation tubes. PPP- platelet-poor plasma.						

### Storage temperature and delay: between-group analyses

The 15 miRNAs undetected in the samples immediately frozen at - 80°C were also undetected in all the samples stored for 24h at RT and 4°C.

Considerable differences in the number of undetected miRNAs were observed in the sample stored for 24h at either RT and 4°C considering the different collection conditions (Table 3, Supplementary Table 2 and 4), with the exception of samples stored for 24h at 4°C collected in K2EDTA and PPT tubes. Fold change analyses showed 2% up-regulated and 12% down-regulated miRNAs in PPP samples compared to the K2EDTA stored for 24h at RT and 1% up-regulated and 5% down-regulated miRNAs in PPP samples compared to the K2EDTA samples stored for 24h at 4°C (Table 2). No differences in miRNA expression were observed between PPT and K2EDTA samples stored for 24h at RT, whereas after storage for 24h at 4°C, 1% of miRNAs were upregulated in the PPT compared to the K2EDTA samples (Table 2).

### Storage temperature and delay: within-group analyses

Analysis of miRNAs detectability revealed significant differences between samples immediately frozen at - 80°C, and stored for 24h at either RT or 4°C, collected in both K2EDTA and PPP (Table 4, Supplementary Tables 3 and 5). On the contrary, no differences in miRNA detectability emerged when comparing different storage conditions for samples collected in PPT (Table 4, Supplementary Tables 3 and 5).

**Table 4:** Within-group comparison of detected and undetected miRNAs

**Storage condition**	**miRNAs**	**- 80°C, N (%)** **N = 179**	**RT 24h, N (%)** **N = 179**	**24h 4°C, N (%)** **N = 179**	**χ^2^ Test**	**P**
K2EDTA	detected	96 (54)	136 (76)	159 (89)	57.36	< 0.001
	undetected	83 (46)	43 (24)	20 (11)		
PPP	detected	103 (58)	73 (41)	75 (42)	12.63	0.002
	undetected	76 (42)	106 (59)	104 (58)		
PPT	detected	161 (90)	155 (87)	157 (88)	0.99	0.608
	undetected	18 (10)	24 (13)	22 (12)		
Pearson’s Chi-Square test with Yates’ continuity correction was performed for detected and undetected miRNAs from samples collected in K2EDTA, PPP, and PPT among different storage conditions (immediately stored at - 80°C, stored for 24h at RT, and stored for 24h at 4°C). P < 0.05 was considered statistically significant. RT – room temperature. K2EDTA - dipotassium ethylendiaminetetraacetate tubes. PPT – plasma preparation tubes. PPP- platelet-poor plasma.						

Analysis of FC revealed that the percentage of up- and down-regulated miRNAs after storage for 24h at RT, compared with samples immediately frozen at - 80°C, were, respectively, 5% and 4% for K2EDTA samples, 2% and 1% for PPP samples, 1% and 0% for PPT samples. Compared with samples immediately frozen at - 80°C, the results after plasma sample storage for 24h at 4°C showed 1% up-regulated and 1% down-regulated miRNAs in K2EDTA samples, 0% up-regulated and 1% down-regulated miRNA in PPP samples, and 0% up-regulated and 1% down-regulated miRNA in 1 PPT samples (Table 5).

**Table 5:** Within-group comparison of circulating miRNA expression

	**Group**											
**miRNAs,**	**K2EDTA**				**PPP**				**PPT**			
**fold change (P)**	**24h RT *vs* - 80°C**	**24h 4°C *vs* - 80°C**	**24h 4°C *vs* 24h RT**	**P**	**24h RT *vs* - 80°C**	**24h 4°C *vs* - 80°C**	**24h 4°C *vs* 24h RT**	**P**	**24h RT *vs* - 80°C**	**24h 4°C *vs* - 80°C**	**24h 4°C *vs* 24h RT**	**P**
hsa-miR-106b-3p	1.34	58.43	43.52	0.058	0.74	0.74	1.00	0.723	1.46 (0.890)	0.04 (0.408)	0.03 (0.034)	0.039
hsa-miR-10b-5p	0.78	0.92	1.17	0.782	253.03 (0.022)	186.35 (0.539)	0.74 (0.539)	0.027	18.71 (0.303)	32.18 (0.051)	1.72 (0.990)	0.051
hsa-miR-125a-5p	1.25	0.47	0.37	0.099	123.58 (0.022)	58.10 (0.539)	0.47 (0.539)	0.027	1.66	1.29	0.77	0.148
hsa-miR-125b-5p	0.005 (0.022)	0.21 (0.539)	40.46 (0.539)	0.027	0.89	0.89	1.00	0.965	0.51 (0.539)	0.03 (0.022)	0.05 (0.539)	0.027
hsa-miR-142-3p	0.14 (0.034)	0.31 (0.408)	2.13 (0.890)	0.039	0.55 (0.990)	0.24 (0.051)	0.44 (0.303)	0.051	0.33 (0.051)	0.77 (0.990)	2.34 (0.303)	0.051
hsa-miR-143-3p	1.00	74.97	74.97	0.063	0.01 (0.470)	0.01 (0.027)	0.65 (0.699)	0.032	0.84	2.15	2.57	0.177
hsa-miR-148b-3p	1.00	158.27	158.27	0.063	1.61 (0.539)	0.01 (0.539)	0.003 (0.022)	0.027	1.54	1.29	0.84	0.875
hsa-miR-15b-3p	58.60 (0.408)	85.52 (0.034)	1.46 (0.890)	0.039	1.01	150.74	149.81	0.064	0.50	0.72	1.43	0.079
hsa-miR-19a-3p	0.16 (0.022)	0.39 (0.539)	2.43 (0.539)	0.027	1.04	2.37	2.27	0.393	0.95	4.36	4.59	0.061
hsa-miR-20a-5p	0.30 (0.022)	0.49 (0.539)	1.65 (0.539)	0.027	0.30	0.93	3.10	0.061	1.47	1.97	1.33	0.202
hsa-miR-215-5p	0.005 (0.022)	0.38 (0.539)	80.98 (0.539)	0.027	0.00	1.08	227.26	0.061	0.44	0.69	1.59	0.329
hsa-miR-223-3p	1.65 (0.022)	2.44 (0.539)	1.47 (0.539)	0.027	0.18 (0.022)	0.59 (0.539)	3.25 (0.539)	0.027	1.68	1.52	0.91	0.059
hsa-miR-29b-3p	1.26 (0.699)	90.01 (0.027)	71.33 (0.470)	0.032	1.13	1.13	1.00	0.723	0.03	0.03	1.00	0.063
hsa-miR-29c-3p	105.48	155.82	1.48	0.061	0.00	0.00	1.00	0.063	0.02 (0.408)	2.10 (0.890)	116.47 (0.034)	0.039
hsa-miR-30a-5p	0.94	43.97	46.60	0.064	361.27 (0.022)	213.25 (0.539)	0.59 (0.539)	0.027	1.11	1.46	1.31	0.733
hsa-miR-324-3p	82.55 (0.022)	45.45 (0.539)	0.55 (0.539)	0.027	1.02	1.02	1.00	0.965	0.02	0.02	1.00	0.063
hsa-miR-32-5p	0.84 (0.539)	0.02 (0.022)	0.02 (0.539)	0.027	0.01	0.01	1.10	0.064	25.95 (0.042)	6.99	0.27	0.047
hsa-miR-335-3p	109.94 (0.022)	40.61 (0.539)	0.37 (0.539)	0.027	0.86	0.86	1.00	0.723	0.70	1.10	1.56	0.329
hsa-miR-376a-3p	119.83 (0.022)	60.29 (0.539)	0.50 (0.539)	0.027	1.10	1.06	0.96	0.243	1.45	1.12	0.77	0.733
hsa-miR-382-5p	48.84 (0.034)	34.56 (0.408)	0.71 (0.890)	0.039	1.00	1.00	1.00	1.000	1.30	1.19	0.91	0.733
hsa-miR-424-5p	0.01 (0.022)	0.42 (0.539)	53.00 (0.539)	0.027	291.39	1.00	0.00	0.063	26.76	1.00	0.04	0.063
	**Group**											
**miRNAs,**	**K2EDTA**				**PPP**				**PPT**			
**fold change (P)**	**24h RT *vs* - 80°C**	**24h 4°C *vs* - 80°C**	**24h 4°C *vs* 24h RT**	**P**	**24h RT *vs* - 80°C**	**24h 4°C *vs* - 80°C**	**24h 4°C *vs* 24h RT**	**P**	**24h RT *vs* - 80°C**	**24h 4°C *vs* - 80°C**	**24h 4°C *vs* 24h RT**	**P**
hsa-miR-425-3p	70.16 (0.022)	36.94 (0.539)	0.53 (0.539)	0.027	0.93	0.93	1.00	0.723	1.46	0.95	0.65	0.193
hsa-miR-454-3p	85.10 (0.034)	48.15 (0.408)	0.57 (0.890)	0.039	0.91	0.91	1.00	0.723	0.85	0.61	0.72	0.252
hsa-miR-485-3p	102.79 (0.034)	52.17 (0.408)	0.51 (0.890)	0.039	1.00	1.00	1.00	1.000	0.88	0.01	0.02	0.061
hsa-miR-574-3p	0.30 (0.034)	0.54 (0.408)	1.81 (0.890)	0.039	0.02 (0.470)	0.01 (0.027)	0.80 (0.699)	0.032	0.84	0.78	0.93	0.67
hsa-miR-629-5p	0.81	0.89	1.09	0.561	2.91 (0.470)	0.01 (0.699)	0.004 (0.027)	0.032	0.91	1.98	2.18	0.707
hsa-miR-93-3p	70.27	77.79	1.11	0.061	0.01 (0.539)	1.54 (0.539)	156.47 (0.022)	0.027	0.46	0.76	1.64	0.561
hsa-miR-99a-5p	93.07 (0.034)	48.88 (0.408)	0.53 (0.890)	0.039	0.87	1.00	1.15	0.723	38.95	33.56	0.86	0.061
Expressions of miRNAs for each collection condition (K2EDTA, PPP, and PPT) from samples immediately stored at - 80°C, and stored for 24h at RT or 4°C were compared with Kruskal-Wallis test. P < 0.05 was considered statistically significant. Dunn’s multiple comparison test was performed for comparison between two storage conditions, only for significant Kruskal-Wallis test. Differences in miRNA expression between two conditions is reported as the ratio of miRNA expression of an “experimental” condition and a “reference” condition (fold change). Results are reported only for miRNAs with a statistically significant ≥ ± 5-fold change, in at least one comparison. RT – room temperature. K2EDTA - dipotassium ethylendiaminetetraacetate tubes. PPT – plasma preparation tubes. PPP- platelet-poor plasma.												

### Circulating miRNA expression quantification

Among clinically relevant miRNAs associated with skeletal muscle, bone, and cancer from K2EDTA samples (hsa-miR-125b-5p, hsa-miR-29b-3p, and hsa-miR-376a-3p), PPP samples (hsa-miR-125a-5p), and PPT samples (hsa-miR-125b-5p), the circulating profile in the three storage conditions (- 80°C, 24h RT, and 24h 4°C) differed significantly (Table 5, Supplementary Table 6).

Some clinically relevant miRNAs were down-regulated in PPP samples compared to the other collection conditions. Specifically, hsa-miR-125b-5p, hsa-miR-152-3p, hsa-miR-375 were lower in PPP samples compared to K2EDTA, while hsa-miR-378a-3p compared to PPT samples, when immediately stored at - 80°C. When stored for 24h at RT, hsa-miR-1, hsa-miR-195a-5p, hsa-miR-375 were down-regulated in PPP samples compared to K2EDTA sample, and hsa-miR-378a-3p compared to PPT samples. Similarly, hsa-miR-1 was lower in PPP compared to K2EDTA samples, and hsa-miR-375 compared to PPT samples when stored for 24h at 4°C (Table 2, Supplementary Table 1). Despite platelet depletion, some miRNAs were actually up-regulated in the PPP as compared to the PPT (hsa-miR-152-3p and hsa-miR-378-3p) and to K2EDTA samples (hsa-miR-210-3p) (Table 2, Supplementary Table 1).

Noteworthy, the miRNA expression in the PPT samples were generally less affected by storage condition due to the lowest FC values for PPT samples (Table 5, Supplementary Table 6).

## Discussion

Several preanalytical variables are thought to influence the quantitation of circulating miRNAs. Here, we provide an analysis of four preanalytical variables (collection matrix, platelet depletion, storage delay time and storage delay temperature) that could affect plasma circulating miRNA stability and, thus, miRNA detection and quantitation.

Regarding the sample matrix, EDTA has been demonstrated not to interfere with enzyme activities in PCR-based assays and is considered the best anticoagulant for circulating miRNA profiling (*17*). Hence, for our study we chose tubes spray-coated with EDTA either with or without a gel separator (PPT and K2EDTA, respectively).

Several studies have demonstrated that miRNA concentrations in EDTA-plasma samples are influenced by centrifugation time and speed, since platelets and microparticles represent a source of contamination for circulating miRNAs (*24*, *26*). Recently, Basso *et al.* reported that EDTA-, citrate-, acid citrate dextrose-anticoagulated plasma and serum were all comparable and superior to heparin-plasma in terms of miRNA quality. In terms of miRNA yields, EDTA-plasma was affected by storage (over 9h at RT) and double centrifugation. Finally, EDTA-plasma miRNA expression profiles were strongly affected by the centrifugation protocol rather than temperature or time to separation (*16*). In another recent study, by Binderup, the impact of standard centrifugation, single prolonged centrifugation (3000xg for 30 min) and double centrifugation (2x3000xg for 15 min) on plasma miR-92, miR-126, and miR-16 expression was investigated. Differences in miR-92 and miR-126 expression, after single prolonged and double centrifugation, were evidenced when data were normalized on miR-16, miR-16-cel-39, but not when normalized on cel-39. Plasma expression of miR-92 and miR-126 fairly correlated when samples underwent to either standard (2000xg for 10 min) or double centrifugation and normalized on miR-16 (*27*). Besides the preanalytical-related information, this paper further highlights the issue of sample processing (*5*). McDonald and colleagues observed that differential processing alters miRNAs quantitation. An additional centrifugation step in plasma processing to reduce the effects of cellular contaminants significantly influenced miR-15 and miR-24 concentrations, while miR-26 concentration was only slightly reduced (*28*). Accordingly, for our K2EDTA samples, we applied stepwise centrifugation of plasma to assess the contribution of platelet-derived contaminants potentially affecting miRNA quantitation (PPP).

The effect of storage on plasma miRNAs stability remains debated (*14*, *29*). Mitchell *et al.* demonstrated that miRNA expression (miR-15, miR-16, miR-16, and miR-24) were only minimally affected by incubation at RT for 24h, whereas Glinge demonstrated a reduction in plasma miR-1 and miR-21 expression after 24h at RT (*14*, *29*).

To assess the effect of storage conditions on plasma circulating miRNA expression, for our study miRNA expression in plasma samples collected and immediately frozen at -80°C were compared to those collected and frozen with 24h delay stored at either RT or 4°C. This was done to simulate real-world situations in the clinical laboratory. By the end, 179 circulating miRNAs were assayed in nine different conditions: plasma from K2EDTA tubes or PPT tubes immediately frozen at - 80°C or stored for 24h at either RT or at 4°C and plasma from K2EDTA tubes depleted of platelets (PPP) stored as above.

Our results show that each condition was associated with a specific circulating miRNA profile, demonstrating that sample matrix, processing, and storage can all affect the analytical output in circulating miRNA measurement. Analyses of detectability showed that regardless of the storage condition, miRNA detectability in the PPP samples was notably affected (Table 3, Supplementary Tables 2 and 4). The frequency of undetected miRNAs in the K2EDTA compared to PPT samples was significantly reduced in each storage condition, except for 24h at 4°C in which miRNA detectability was comparable (Supplementary Tables 2 and 4). Regardless of the sample matrix, miRNA detectability was less influenced by storage conditions in the PPT samples: no differences of undetected miRNAs were observed (Table 4, Supplementary Table 3 and 5). Contrarily, K2EDTA samples displayed the greatest variability in miRNA detectability (Table 4, Supplementary Table 3 and 5): the percentage of detectable miRNAs increased during storage for 24h, particularly at 4°C. The amount of detectable miRNAs was higher in the PPT samples than in either the K2EDTA or the PPP samples, revealing that gel-mediated separation can actually increase the yield in miRNAs by contemporarily keeping cell contamination low.

According to previous studies on plasma processing and platelets contaminant removal (*26*-*28*), the stepwise centrifugation in K2EDTA samples led to reduced expression of platelet-derived miRNAs (*24*, *25*), and also of other miRNAs of other cellular origin (*16*). This reduction was more evident after storage for 24h, highlighting a consistent instability of miRNAs in the PPP samples as compared to miRNA in the K2EDTA samples. The miRNA expression were higher in the PPT than the PPP tubes, while they were comparable in the PPT and the K2EDTA tubes when the plasma samples were immediately frozen at -80°C. Analyses of FC showed that miRNAs in the PPT samples were less influenced by temperature and length of storage: the PPT samples had the lowest number of modulated (up- and down-regulated) miRNAs. These results indicate that the PPT samples, possibly thanks to the low cellularity in the plasma due to gel-mediated separation, have greater stability of circulating miRNAs in different storage conditions, *e.g.*, those found in routine laboratory practice. Hence, PPT plasma samples could be considered a suitable matrix for miRNA measurement.

Analyses of single miRNAs of clinical relevance (*e.g.*, associated with skeletal muscle, bone, and cancer) shared the previous results about the greater stability of miRNA from samples collected in the PPT tubes, regardless of storage length and temperature. Notably, it emerged that each miRNA had a different circulating profile in each condition. This is of clinical relevance because a combination of various conditions could have a profound effect on analytical output and, hence, give rise to misinterpretation of results. Furthermore, our analysis showed that different miRNAs are differently affected by the same collection method or storage condition. This represents a great limitation for studies of pre-analytical variables, where few miRNAs are analysed, since a limited number of miRNAs cannot be representative of all circulating miRNAs. The analysis of a large amount of miRNAs, as in the present study, may offer a better overview and a more detailed description of the effects of different pre-analytical variables on the measurability of circulating miRNAs.

The main limitation of the present study is the limited number of variables that were considered. Indeed, given a potential list of preanalytical variables that can affect analytical output, here we just focused on only a few. Nonetheless, our study addresses part of most complex subject that needs further investigation. Furthermore, the rigorous experimental design provides reasonable certainty about the practical utility of the results reported here.

Circulating miRNAs are emerging as potential biomarkers for diagnostic, prognostic, and predictive purposes; meanwhile, there is a growing interest in the preanalytical variables that could affect their quantitation (*2*, *6*). Standardization of these variables represents a critical step in the clinical implementation of miRNAs, since, as shown in this study, variables such as matrix, platelet depletion, and storage conditions can all alter miRNA measurement.

## Supplementary material

010703_ Supplementary tables
