# Supplementary material for: Study of the preanalytical variables affecting the measurement of clinically relevant free-circulating microRNAs: focus on sample matrix, platelet depletion, and storage conditions
Source: Biochem Med (Zagreb). 2019 Dec 15;30(1):010703. doi: 10.11613/BM.2020.010703 (PMC6904965; doi:10.11613/BM.2020.010703)
Supplement: Supplementary file 1 — 010703_ Supplementary tables [file bm-30-1-010703-S1.pdf]

Supplementary Table 1. Complete between-group comparison of circulating miRNA expression

miRNAs,	Storage condition											
	Immediately frozen at - 80°C				24h RT				24h 4°C			
fold change (P)	PPT *vs* K2EDTA	K2EDTA *vs* PPP	PPT *vs* PPP	P	PPP *vs* K2EDTA	PPT *vs* K2EDTA	PPT *vs* PPP	P	PPP *vs* K2EDTA	PPT *vs* K2EDTA	PPT *vs* PPP	P
hsa-let-7a-5p	1.90 (0.408)	1.72 (0.890)	3.26 (0.034)	0.039	0.24	1.10	0.22	0.061	0.43	0.93	1.92	0.067
hsa-let-7b-3p	35.73	1.00	35.73	0.063	0.77	13.21	0.06	0.058	0.85	1.10	14.55	0.782
hsa-let-7b-5p	2.29 (0.303)	0.33 (0.051)	0.76 (0.990)	0.051	0.50 (0.034)	0.81 (0.890)	0.62 (0.408)	0.039	1.35	1.14	2.18	0.148
hsa-let-7c-5p	1.48	1.10	1.62	0.733	0.01 (0.051)	0.68 (0.990)	0.01 (0.303)	0.051	0.02	0.77	1.71	0.061
hsa-let-7d-3p	0.61 (0.303)	2.62 (0.051)	1.59 (0.990)	0.051	0.77	1.59	0.48	0.079	1.04	1.35	2.16	0.561
hsa-let-7d-5p	2.87 (0.051)	0.78 (0.990)	2.24 (0.303)	0.051	0.01	1.00	0.01	0.067	0.65	1.38	64.99	0.301
hsa-let-7e-5p	59.26	0.01	0.80	0.067	0.01	1.33	0.01	0.067	0.97	0.59	89.87	0.288
hsa-let-7f-5p	1.25	2.04	2.56	0.067	0.35	0.99	0.35	0.067	0.53	1.17	1.51	0.301
hsa-let-7g-5p	1.08	1.03	1.12	0.875	0.72	0.72	1.01	0.393	0.52	0.42	0.51	0.079
hsa-let-7i-5p	1.12	0.83	0.93	0.733	0.78	0.71	1.11	0.393	1.71	0.87	1.54	0.393
hsa-miR-1	12.84	0.02	0.29	0.092	**0.02 (0.027)**	0.12 (0.470)	0.16 (0.699)	0.032	**0.02 (0.027)**	0.20 (0.470)	0.11 (0.699)	0.032
hsa-miR-101-3p	0.25	3.32	0.82	0.061	1.79	0.72	2.49	0.252	0.87	0.48	0.35	0.288
hsa-miR-103a-3p	0.81 (0.991)	3.80 (0.051)	3.07 (0.303)	0.051	1.02	2.22	0.46	0.061	0.65	1.22	1.40	0.491
hsa-miR-106a-5p	1.36	1.78	2.41	0.051	0.60 (0.539)	3.62 (0.539)	**0.17 (0.022)**	0.027	0.73	0.58	4.41	0.079
hsa-miR-106b-3p	36.28	0.74	27.02	0.058	0.74	39.55	0.02	0.058	0.02	0.02	0.91	0.058
hsa-miR-106b-5p	0.56	0.80	0.44	0.393	0.01 (0.051)	0.53 (0.990)	0.01 (0.303)	0.051	1.25	0.61	103.59	0.393
hsa-miR-107	0.66	2.00	1.32	0.561	0.64	0.60	1.08	0.875	0.65	1.25	0.61	0.561

hsa-miR-10b-5p	4.36	1.00	4.36	1.000	**323.51 (0.022)**	23.92 (0.539)	13.52 (0.539)	0.027	**203.30 (0.022)**	35.11 (0.539)	15.03 (0.539)	0.027
hsa-miR-122-5p	0.80	0.37	0.30	0.061	3.31 (0.539)	0.34 (0.539)	**9.71 (0.022)**	0.027	2.22 (0.539)	0.33 (0.539)	0.023 (0.022)	0.027
hsa-miR-125a-5p	0.77 (0.990)	96.22 (0.051)	74.14 (0.303)	0.051	1.03	1.03	1.00	0.957	1.29	2.12	1.29	0.061
hsa-miR-125b-5p	0.22 (0.539)	**169.01 (0.022)**	36.73 (0.539)	0.027	1.00	21.01	0.05	0.063	0.02	0.03	0.52	0.064
hsa-miR-1260a	43.13	0.94	40.37	0.058	**0.01 (0.022)**	0.42 (0.539)	0.03 (0.539)	0.027	**0.01 (0.022)**	0.79 (0.539)	0.58 (0.539)	0.027
hsa-miR-126-3p	1.59 (0.539)	2.78 (0.539)	**4.42 (0.022)**	0.027	0.64 (0.539)	1.83 (0.539)	**0.35 (0.022)**	0.027	0.53 (0.539)	1.63 (0.539)	**1.53 (0.022)**	0.027
hsa-miR-126-5p	1.53	0.75	1.15	0.561	0.75	1.90	0.39	0.061	0.52 (0.408)	2.82 (0.899)	1.32 (0.038)	0.039
hsa-miR-127-3p	34.15	1.00	34.15	0.063	0.02	1.68	0.01	0.077	0.02	0.98	1.69	0.067
hsa-miR-128-3p	1.00	1.12	1.12	0.869	1.01	53.70	0.02	0.064	**0.02 (0.028)**	0.71 (0.539)	1.17 (0.539)	0.027
hsa-miR-130a-3p	34.02	1.60	54.28	0.058	0.02	1.18	0.02	0.067	0.02	1.26	1.06	0.067
hsa-miR-130b-3p	1.32	0.76	1.00	0.723	1.66	2.19	0.76	0.243	0.07	0.09	0.09	0.097
hsa-miR-132-3p	32.00	0.92	29.50	0.058	0.01 (0.051)	0.40 (0.990)	0.02 (0.303)	0.051	0.02 (0.303)	1.61 (0.990)	1.15 (0.051)	0.051
hsa-miR-133b	1.75	0.77	1.35	0.243	1.30	1.75	0.74	0.243	0.74	26.98	1.00	0.058
hsa-miR-139-5p	1.13	66.55	75.01	0.610	0.02	1.08	0.01	0.067	0.02 (0.051)	0.75 (0.990)	1.30 (0.051)	0.051
hsa-miR-140-3p	0.66	0.83	0.55	0.670	1.62 (0.539)	0.50 (0.539)	3.24 (0.022)	0.027	1.10	0.38	0.34	0.067
hsa-miR-140-5p	41.24	1.00	41.24	0.063	1.00	32.90	0.03	0.063	1.10 (0.699)	**41.32 (0.027)**	36.22 (0.470)	0.032
hsa-miR-142-3p	1.02	5.39	5.49	0.067	0.71	2.32	0.31	0.099	0.15 (0.539)	2.55 (0.539)	0.48 (0.022)	0.027
hsa-miR-142-5p	0.92	179.70	165.69	0.061	0.01	1.61	0.01	0.061	0.03	3.25	4.67	0.067
hsa-miR-143-3p	34.31 (0.539)	**0.01 (0.022)**	0.22 (0.539)	0.027	1.38	28.73	0.05	0.055	0.01	0.98	0.25	0.067

hsa-miR-144-3p	**0.17 (0.034)**	1.74 (0.890)	0.29 (0.408)	0.039	6.30	0.51	12.43	0.148	2.31	1.63	0.19	0.491
hsa-miR-144-5p	53.84	1.04	56.03	0.064	**0.01 (0.034)**	0.45 (0.890)	0.02 (0.408)	0.039	1.62 (0.539)	0.27 (0.539)	**76.36 (0.022)**	0.027
hsa-miR-145-5p	0.79	1.69	1.33	0.113	1.09	1.00	1.09	0.733	1.27	1.21	1.16	0.670
hsa-miR-146a-5p	1.21	2.81	3.40	0.061	0.75 (0.990)	2.52 (0.303)	0.30 (0.022)	0.027	0.66	1.82	2.19	0.148
hsa-miR-146b-5p	32.94 (0.539)	**0.01 (0.022)**	0.26 (0.539)	0.027	**0.01 (0.022)**	0.49 (0.539)	0.02 (0.539)	0.027	0.01	0.83	0.63	0.061
hsa-miR-148a-3p	108.34	1.00	108.34	0.063	0.01	0.96	0.01	0.061	0.01	0.97	0.92	0.061
hsa-miR-148b-3p	108.18	0.01	0.58	0.061	298.06	166.42	1.79	0.061	0.01	0.88	0.00	0.067
hsa-miR-150-5p	0.59 (0.539)	0.56 (0.539)	0.33 (0.022)	0.027	1.99	0.40	5.01	0.079	2.71	0.90	0.54	0.067
hsa-miR-151a-3p	2.50	1.09	2.74	0.430	0.74	1.76	0.42	0.202	0.02 (0.303)	1.69 (0.990)	0.04 (0.051)	0.051
hsa-miR-151a-5p	1.03	2.35	2.42	0.193	0.85	2.24	0.38	0.061	0.62	2.34	1.65	0.058
hsa-miR-152-3p	0.47 (0.539)	**106.31 (0.022)**	50.45 (0.539)	0.027	1.81	1.03	1.75	0.067	0.02 (0.408)	2.70 (0.890)	**0.01 (0.034)**	0.039
hsa-miR-154-5p	43.89	0.87	38.31	0.058	0.80	29.02	0.03	0.058	1.13	1.41	41.02	0.634
hsa-miR-155-5p	56.78 (0.408)	**0.01 (0.034)**	0.41 (0.890)	0.039	0.01	0.61	0.02	0.067	1.10	67.05	62.57	0.064
hsa-miR-15a-5p	1.97 (0.539)	**0.20 (0.022)**	0.39 (0.539)	0.027	2.40	0.81	2.95	0.061	1.21	0.43	0.41	0.061
hsa-miR-15b-3p	63.95	1.00	63.95	0.063	**0.02 (0.034)**	0.55 (0.890)	0.03 (0.408)	0.039	1.76 (0.539)	0.54 (0.539)	**56.45 (0.022)**	0.027
hsa-miR-15b-5p	0.85	1.08	0.92	0.837	0.02	1.50	0.01	0.059	0.01	1.56	1.16	0.061
hsa-miR-16-2-3p	45.83 (0.539)	**0.01 (0.022)**	0.26 (0.539)	0.027	0.01	0.01	1.00	0.063	0.01	0.02	0.01	0.058
hsa-miR-16-5p	0.89 (0.539)	0.48 (0.539)	0.43 (0.022)	0.027	2.86 (0.408)	0.83 (0.890)	3.46 (0.034)	0.039	3.31 (0.028)	1.46 (0.539)	0.96 (0.539)	0.027
hsa-miR-17-5p	37.86	0.98	37.26	0.064	1.00	32.00	0.03	0.063	0.02 (0.408)	1.38 (0.890)	0.52 (0.034)	0.039
hsa-miR-181a-5p	1.75	1.57	2.76	0.061	0.02	1.47	0.01	0.067	0.03	0.95	2.36	0.236
hsa-miR-185-5p	0.38 (0.539)	0.83 (0.539)	0.31 (0.022)	0.027	0.44	0.72	0.61	0.051	2.31	2.00	3.76	0.193

hsa-miR-186-5p	1.21	0.83	1.00	0.965	**0.01 (0.022)**	0.35 (0.539)	0.03 (0.539)	0.027	0.58	1.21	21.01	0.634
hsa-miR-18a-5p	0.63	1.44	0.91	0.061	0.02	0.74	0.02	0.061	0.02	0.94	0.78	0.061
hsa-miR-18b-5p	0.51 (0.990)	150.93 (0.051)	77.03 (0.303)	0.051	0.02	0.95	0.02	0.061	0.01	0.94	0.72	0.067
hsa-miR-191-5p	1.94 (0.890)	3.57 (0.408)	**6.94 (0.034)**	0.039	0.16	1.15	0.14	0.051	0.34 (0.890)	1.67 (0.408)	2.42 (0.034)	0.039
hsa-miR-192-5p	0.50 (0.539)	0.44 (0.539)	0.22 (0.022)	0.027	3.98	0.74	5.39	0.051	1.80 (0.890)	0.39 (0.408)	0.33 (0.038)	0.039
hsa-miR-193a-5p	32.75	0.98	25.52	0.058	0.79	1.34	0.59	0.243	0.78	1.00	1.32	0.723
hsa-miR-194-5p	28.90 (0.539)	**0.01 (0.022)**	0.16 (0.539)	0.027	5.78 (0.539)	0.39 (0.539)	**14.87 (0.022)**	0.027	2.63 (0.539)	0.53 (0.539)	**0.18 (0.022)**	0.027
hsa-miR-195-5p	46.57	1.00	46.57	0.063	**0.01 (0.022)**	0.54 (0.539)	0.03 (0.539)	0.027	0.01 (0.303)	2.48 (0.990)	0.34 (0.051)	0.051
hsa-miR-197-3p	0.93	1.13	1.06	0.875	2.11	1.43	1.48	0.491	1.42	1.10	0.96	0.561
hsa-miR-199a-3p	1.46	41.25	60.27	0.061	0.01	1.65	0.01	0.061	1.23	0.85	153.60	0.252
hsa-miR-199a-5p	0.46 (0.539)	**60.84 (0.022)**	28.07 (0.539)	0.027	0.87	48.86	0.02	0.064	0.87	49.74	48.86	0.064
hsa-miR-19a-3p	0.18	5.25	0.94	0.061	1.23	1.06	1.16	0.733	1.15	2.00	0.99	0.099
hsa-miR-19b-3p	0.27 (0.051)	2.62 (0.303)	0.71 (0.909)	0.051	2.14	1.36	1.57	0.099	0.51	0.36	0.32	0.061
hsa-miR-200c-3p	33.46	0.78	26.21	0.058	0.01 (0.051)	0.48 (0.990)	0.02 (0.303)	0.051	0.03	1.61	1.13	0.141
hsa-miR-205-5p	7.69	0.01	0.08	0.092	1.00	6.38	0.16	0.106	2.41	0.15	15.36	0.241
hsa-miR-20a-5p	0.34 (0.539)	1.65 (0.539)	0.56 (0.022)	0.027	0.62 (0.408)	1.68 (0.890)	0.37 (0.034)	0.039	1.16	1.36	3.17	0.252
hsa-miR-210-3p	33.68 (0.539)	**0.005 (0.022)**	0.16 (0.539)	0.027	1.00	1.17	0.85	0.723	0.02	0.02	0.02	0.058
hsa-miR-2110	42.84	0.01	0.52	0.082	**0.01 (0.034)**	0.40 (0.890)	0.03 (0.408)	0.039	**0.01 (0.022)**	0.48 (0.539)	0.44 (0.539)	0.027
hsa-miR-215-5p	0.36 (0.303)	0.87 (0.990)	0.31 (0.051)	0.051	1.16	33.39	0.03	0.064	3.25 (0.539)	0.66 (0.539)	**93.70 (0.022)**	0.027
hsa-miR-21-5p	1.61	1.08	1.75	0.067	1.20	1.17	1.02	0.202	0.20 (0.539)	1.64 (0.539)	**0.19 (0.022)**	0.027
hsa-miR-221-3p	0.42	3.87	1.62	0.058	1.06	2.33	0.46	0.193	1.28	1.42	2.81	0.670

hsa-miR-222-3p	0.96	0.41	0.40	0.061	5.70	0.95	6.02	0.067	2.33	1.12	0.39	0.193
hsa-miR-223-3p	1.53 (0.539)	1.61 (0.539)	2.48 (0.022)	0.027	0.07 (0.539)	1.55 (0.539)	**0.04 (0.022)**	0.027	0.15	0.95	3.42	0.067
hsa-miR-223-5p	38.34	1.00	38.34	0.063	1.00	42.19	0.02	0.063	0.02	0.02	0.67	0.058
hsa-miR-22-3p	0.59	0.71	0.42	0.202	3.47	1.39	2.49	0.193	2.38	1.36	0.96	0.193
hsa-miR-23a-3p	1.71 (0.539)	1.66 (0.539)	2.85 (0.022)	0.027	0.36	1.84	0.19	0.061	0.43 (0.539)	1.37 (0.539)	2.23 (0.022)	0.027
hsa-miR-23b-3p	2.22 (0.990)	1.54 (0.303)	3.41 (0.051)	0.051	0.52 (0.303)	1.27 (0.990)	0.41 (0.051)	0.051	0.20 (0.539)	1.63 (0.539)	0.50 (0.022)	0.027
hsa-miR-24-3p	1.54 (0.539)	3.51 (0.539)	**5.39 (0.022)**	0.027	0.20 (0.303)	1.12 (0.990)	0.18 (0.051)	0.051	0.66 (0.539)	2.45 (0.539)	3.67 (0.022)	0.027
hsa-miR-25-3p	0.76 (0.539)	0.64 (0.539)	0.49 (0.022)	0.027	1.64 (0.539)	0.53 (0.539)	3.08 (0.022)	0.027	1.78 (0.408)	0.81 (0.890)	0.58 (0.034)	0.039
hsa-miR-26a-5p	1.31	2.02	2.65	0.202	0.50 (0.408)	1.58 (0.890)	0.32 (0.034)	0.039	0.43 (0.539)	3.16 (0.539)	1.37 (0.022)	0.027
hsa-miR-26b-5p	0.48	1.05	0.51	0.177	1.19	0.85	1.39	0.558	0.03	1.53	0.02	0.065
hsa-miR-27a-3p	0.88	0.85	0.74	0.491	0.03	1.20	0.02	0.061	0.02	1.33	0.98	0.061
hsa-miR-27b-3p	0.63 (0.408)	2.58 (0.034)	1.63 (0.890)	0.039	0.63	1.59	0.40	0.058	0.59	1.40	1.48	0.079
hsa-miR-28-3p	71.88	1.00	71.88	0.063	0.02 (0.303)	1.46 (0.990)	0.02 (0.051)	0.051	0.02 (0.303)	1.69 (0.990)	1.31 (0.051)	0.051
hsa-miR-28-5p	1.04	55.95	58.13	0.061	0.01	0.84	0.02	0.067	0.02	1.09	1.42	0.067
hsa-miR-29a-3p	0.47 (0.539)	0.76 (0.539)	0.35 (0.022)	0.027	6.55	0.93	7.04	0.061	1.79	0.73	0.25	0.099
hsa-miR-29b-3p	37.76	1.00	37.76	0.063	0.89	0.79	1.13	0.243	0.01	0.01	0.01	0.058
hsa-miR-29c-3p	55.34 (0.539)	**0.004 (0.022)**	0.21 (0.539)	0.027	0.01	0.01	1.00	0.063	0.01	0.75	0.01	0.061
hsa-miR-301a-3p	0.36 (0.539)	**170.97 (0.022)**	61.12 (0.539)	0.027	**0.01 (0.034)**	0.71 (0.890)	0.02 (0.408)	0.039	0.02 (0.539)	2.54 (0.539)	1.13 (0.022)	0.027
hsa-miR-30a-5p	34.37	1.07	36.71	0.058	**358.44 (0.022)**	40.39 (0.539)	8.87 (0.539)	0.027	4.54	1.14	0.51	0.061
hsa-miR-30b-5p	1.18	1.59	1.88	0.288	0.63	1.24	0.51	0.099	0.82	0.85	1.60	0.957
hsa-miR-30c-5p	1.17	3.19	3.75	0.061	0.34 (0.034)	0.81 (0.890)	0.41 (0.408)	0.039	0.71	1.17	1.73	0.393

hsa-miR-30d-5p	1.53	1.08	1.65	0.067	0.80 (0.890)	1.62 (0.408)	0.49 (0.034)	0.039	1.08	1.73	2.18	0.301
hsa-miR-30e-3p	38.32 (0.539)	**38.32 (0.022)**	0.33 (0.539)	0.027	**0.01 (0.034)**	0.46 (0.890)	0.02 (0.408)	0.039	0.02	1.75	0.72	0.061
hsa-miR-30e-5p	0.77	1.14	0.87	0.561	0.91	1.07	0.85	0.837	1.04	0.90	1.22	0.957
hsa-miR-320a	1.92 (0.022)	0.64 (0.539)	1.23 (0.539)	0.027	1.79 (0.408)	0.91 (0.890)	1.97 (0.034)	0.039	3.20 (0.022)	1.22 (0.539)	1.63 (0.539)	0.027
hsa-miR-320b	1.96	0.35	0.68	0.079	0.92	0.76	1.21	0.561	3.59 (0.022)	1.32 (0.539)	2.97 (0.539)	0.027
hsa-miR-320c	1.09	0.58	0.63	0.561	1.23	0.72	1.72	0.113	1.59	1.10	0.92	0.193
hsa-miR-320d	0.64 (0.539)	0.68 (0.539)	0.44 (0.022)	0.027	2.55 (0.408)	0.66 (0.890)	3.85 (0.034)	0.039	2.65	1.57	0.69	0.079
hsa-miR-324-3p	49.34	1.00	49.34	0.063	0.01	0.01	1.02	0.064	0.02	0.02	0.02	0.064
hsa-miR-324-5p	82.82	1.15	95.35	0.055	0.89	68.06	0.01	0.058	0.89	64.05	68.06	0.058
hsa-miR-32-5p	0.02 (0.539)	0.47 (0.539)	**0.01 (0.022)**	0.027	**0.02 (0.022)**	0.53 (0.539)	0.04 (0.539)	0.027	1.10	6.99	28.49	0.107
hsa-miR-326	28.51	0.01	0.31	0.092	0.02	0.61	0.03	0.082	0.03	0.70	0.90	0.105
hsa-miR-328-3p	0.71 (0.990)	74.23 (0.051)	52.99 (0.303)	0.051	0.01	0.53	0.02	0.067	0.01 (0.051)	0.78 (0.990)	0.72 (0.408)	0.051
hsa-miR-331-3p	**0.01 (0.034)**	1.64 (0.890)	0.01 (0.408)	0.039	0.02	0.95	0.02	0.061	0.03	1.63	1.76	0.061
hsa-miR-335-3p	50.45	0.86	43.19	0.058	**0.01 (0.022)**	0.32 (0.539)	0.03 (0.539)	0.027	0.02	1.36	0.87	0.067
hsa-miR-338-3p	0.19	81.21	15.65	0.092	1.00	75.88	0.01	0.063	0.05	2.16	3.56	0.092
hsa-miR-339-3p	62.92	0.90	56.39	0.058	0.91	41.67	0.02	0.058	0.99	47.66	45.15	0.064
hsa-miR-339-5p	0.58 (0.890)	**119.87 (0.034)**	69.93 (0.408)	0.039	0.01	1.01	0.01	0.067	0.02	1.10	2.22	0.067
hsa-miR-342-3p	0.60	1.17	0.70	0.288	1.41	1.13	1.25	0.079	1.72 (0.539)	0.72 (0.539)	1.38 (0.028)	0.027
hsa-miR-34a-5p	0.20	1.40	0.28	0.55	1.00	6.76	0.15	0.106	1.00	37.05	6.76	0.063
hsa-miR-361-5p	31.50 (0.303)	0.02 (0.051)	0.70 (0.990)	0.051	0.02	1.14	0.02	0.067	0.80	1.05	45.50	0.202
hsa-miR-363-3p	0.45 (0.539)	**138.59 (0.022)**	63.02 (0.539)	0.027	4.91	0.71	6.96	0.061	1.44	0.64	0.21	0.252
hsa-miR-374a-5p	40.32 (0.470)	1.29 (0.699)	**52.14 (0.027)**	0.032	0.01 (0.051)	0.64 (0.990)	0.02 (0.303)	0.051	0.02	1.22	0.69	0.067

hsa-miR-374b-5p	1.22	49.60	60.46	0.061	0.03 (0.539)	2.06 (0.539)	**0.01 (0.022)**	0.027	0.02	0.96	1.45	0.067
hsa-miR-375	0.22 (0.539)	**195.35 (0.022)**	42.75 (0.539)	0.027	**0.01 (0.022)**	0.22 v	0.03 (0.539)	0.027	11.12 (0.539)	0.11 (0.539)	**427.55 (0.028)**	0.027
hsa-miR-376a-3p	66.12	1.00	66.12	0.063	0.01	0.80	0.01	0.061	0.02	1.23	1.53	0.067
hsa-miR-376c-3p	34.09	1.18	40.38	0.058	0.02	0.85	0.02	0.067	0.02	1.18	1.08	0.061
hsa-miR-378a-3p	0.14 (0.539)	0.51 (0.539)	**0.07 (0.022)**	0.027	4.73 (0.539)	0.39 (0.539)	**12.24 (0.022)**	0.027	2.20	0.57	0.18	0.079
hsa-miR-382-5p	37.50	1.10	41.63	0.064	0.02	1.00	0.02	0.067	0.03	1.29	1.41	0.061
hsa-miR-409-3p	**67.32 (0.027)**	0.89 (0.699)	59.60 (0.470)	0.032	0.01	0.43	0.01	0.061	0.02	1.42	1.33	0.067
hsa-miR-421	31.58	1.00	31.58	0.063	**0.01 (0.034)**	0.48 (0.890)	0.03 (0.408)	0.039	**0.02 (0.022)**	0.64 (0.539)	0.63 (0.539)	0.027
hsa-miR-423-3p	183.06	1.00	183.06	0.063	0.004	0.84	0.005	0.061	0.004	0.69	0.84	0.067
hsa-miR-423-5p	1.72	0.54	0.93	0.561	1.67	1.21	1.39	0.587	1.64	0.80	1.18	0.177
hsa-miR-424-5p	0.01	126.48	1.00	0.063	**291.39 (0.022)**	26.76 (0.539)	10.89 (0.539)	0.027	0.02	0.02	0.00	0.063
hsa-miR-425-3p	47.85	0.93	44.29	0.058	0.01	1.00	0.01	0.067	0.03	1.23	1.89	0.061
hsa-miR-425-5p	91.97 (0.408)	**0.01 (0.034)**	0.50 (0.890)	0.039	0.01	0.62	0.01	0.067	1.28	0.89	124.48	0.561
hsa-miR-451a	0.26	1.10	0.28	0.067	2.68	0.54	4.95 (0.022)	0.027	0.88	0.31	0.18	0.067
hsa-miR-454-3p	**49.88 (0.027)**	0.76 (0.699)	37.92 (0.470)	0.032	**0.01 (0.022)**	0.50 (0.539)	0.03 (0.539)	0.027	0.02	0.63	0.88	0.063
hsa-miR-483-5p	39.02 (0.539)	**0.04 (0.022)**	0.14 (0.539)	0.027	2.29 (0.408)	0.27 (0.890)	**8.59 (0.034)**	0.039	0.01	0.96	0.00	0.061
hsa-miR-484	0.31 (0.022)	1.58 (0.539)	0.50 (0.539)	0.027	1.53	0.69	2.23	0.079	1.25	0.67	0.56	0.202
hsa-miR-485-3p	64.07	1.00	64.07	0.063	0.01	0.55	0.02	0.051	0.02	0.02	1.08	0.058
hsa-miR-486-5p	1.59 (0.539)	0.23 (0.022)	0.37 (0.539)	0.027	1.79 (0.539)	0.21 (0.539)	**8.35 (0.022)**	0.027	3.73 (0.408)	0.81 (0.890)	0.45 (0.034)	0.039
hsa-miR-495-3p	4.15	0.04	0.18	0.092	0.03	0.13	0.27	0.292	0.06	0.72	0.22	0.105
hsa-miR-501-3p	1.28	0.78	1.00	0.723	0.01	0.01	0.84	0.064	**0.02 (0.022)**	0.40 (0.539)	0.02 (0.539)	0.027
hsa-miR-502-3p	1.31	1.00	1.31	0.723	0.02	0.03	0.76	0.058	0.76	15.02	1.00	0.058

hsa-miR-505-3p	38.61	1.00	38.61	0.063	**0.01 (0.022)**	0.21 (0.539)	0.02 (0.539)	0.027	0.01	0.01	0.38	0.055
hsa-miR-532-3p	33.02	0.89	29.46	0.058	1.00	60.70	0.02	0.063	0.02	0.02	0.94	0.063
hsa-miR-532-5p	0.43 (0.539)	0.73 (0.539)	0.31 (0.022)	0.027	0.77	13.39	0.06	0.058	2.90	0.99	50.54	0.061
hsa-miR-543	9.03	1.00	9.03	0.106	0.03	1.68	0.02	0.077	0.05	0.79	2.78	0.105
hsa-miR-574-3p	0.75	2.18	1.63	0.148	0.03 (0.303)	2.12 (0.990)	0.01 (0.051)	0.051	0.01	1.09	0.94	0.067
hsa-miR-584-5p	41.79	0.91	37.90	0.058	0.01	0.81	0.02	0.067	0.02	1.21	1.39	0.061
hsa-miR-590-5p	0.53 (0.408)	0.67 (0.890)	0.36 (0.034)	0.039	4.60	1.37	3.36	0.061	2.08	1.12	0.62	0.061
hsa-miR-629-5p	0.20	0.72	0.15	0.55	5.01 (0.539)	0.23 (0.539)	**21.97 (0.022)**	0.027	0.02	0.45	0.00	0.055
hsa-miR-652-3p	0.98	112.99	110.34	0.061	0.01 (0.051)	0.64 (0.303)	0.01 (0.990)	0.051	0.02	1.12	1.82	0.430
hsa-miR-660-5p	1.00	1.00	1.00	1.000	3.45 (0.539)	0.02 (0.539)	**164.86 (0.022)**	0.027	**0.02 (0.022)**	0.54 (0.539)	0.00 (0.539)	0.027
hsa-miR-7-5p	13.02	0.02	0.29	0.092	1.00	6.20	0.16	0.106	**0.02 (0.027)**	0.15	0.15	0.032
hsa-miR-766-3p	0.52 (0.539)	**63.76 (0.022)**	33.10 (0.539)	0.027	**0.01 (0.034)**	0.78 (0.890)	0.02 (0.408)	0.039	0.02 (0.539)	3.35 (0.539)	1.45 (0.022)	0.027
hsa-miR-874-3p	16.39	0.76	12.41	0.058	0.02	0.02	1.00	0.063	0.76	1.00	0.76	0.723
hsa-miR-877-5p	37.53 (0.539)	**0.012 (0.022)**	0.45 (0.539)	0.027	0.74	33.42	0.02	0.063	**0.01 (0.022)**	0.56 (0.539)	0.38 (0.539)	0.027
hsa-miR-885-5p	7.21 (0.699)	**0.01 (0.027)**	0.06 (0.470)	0.032	1.00	6.18	0.16	0.106	0.02	0.59	0.14	0.129
hsa-miR-92a-3p	1.61	0.55	0.89	0.061	2.23	0.98	2.29	0.061	1.62 (0.539)	0.76 (0.539)	0.71 (0.022)	0.027
hsa-miR-92b-3p	32.41	0.77	24.99	0.058	0.01	0.01	0.77	0.058	1.00	39.60	1.30	0.063
hsa-miR-93-3p	78.27	0.01	0.77	0.061	**0.01 (0.034)**	0.52 (0.890)	0.03 (0.408)	0.039	2.01	0.77	73.21	0.061
hsa-miR-93-5p	0.68	1.78	1.20	0.177	0.84	0.86	0.97	0.252	1.37	1.98	1.41	0.079
hsa-miR-99a-5p	1.15	0.87	1.00	0.723	**0.01 (0.022)**	0.48 (0.539)	0.02 (0.539)	0.027	0.02 (0.051)	0.79 (0.990)	1.06 (0.303)	0.051
hsa-miR-99b-5p	1.79 (0.809)	50.77 (0.408)	**90.83 (0.034)**	0.039	0.01	1.37	0.01	0.067	0.03 (0.408)	1.97 (0.809)	3.22 (0.034)	0.039

Expressions for each miRNAs from K2EDTA, PPP and PPT samples and each storage condition were compared through Kruskal-Wallis test. $P < 0.05$ was considered statistically significant. Dunn's multiple comparison test was performed for comparison between two storage conditions, only for significant Kruskal-Wallis test. Differences in miRNA expression between two conditions is reported as the ratio of miRNA expression of an "experimental" condition and a "reference" condition (fold change). Results are reported only

for miRNAs with a statistically significant ≥ ± 5-fold change, in at least one comparison. K2EDTA - dipotassium ethylendiaminetetraacetate tubes. PPT – plasma preparation tubes. PPP- platelet-poor plasma.

Supplementary Table 2. Detected and undetected miRNA comparison between groups

COLLECTION CONDITION (N = 179)	Detected miRNAs, N (%)	Undetected miRNAs, N (%)	χ^2 Test	P
		- 80°C		
K2EDTA	96 (53.63%)	83 (46.37%)	0.41	0.523
PPP	103(57.54%)	76 (42.46%)		
PPP	103(57.54%)	76 (42.46%)	46.87	< 0.001
PPT	161 (89.94%)	18 (10.06%)		
		24h RT		
K2EDTA	136 (75.98%)	43 (24.02%)	5.95	0.015
PPT	155 (86.59%)	24 (13.41%)		
K2EDTA	136 (75.98%)	43 (24.02%)	44.19	< 0.001
PPP	73 (40.78%)	106 (59.22%)		
PPP	73 (40.78%)	106 (59.22%)	79.24	< 0.001
PPT	155 (86.59%)	24 (13.41%)		
		24h 4°C		
K2EDTA	159 (88.83%)	20 (11.17%)	0.03	0.869
PPT	157 (87.71%)	22 (12.29%)		
K2EDTA	159 (88.83%)	20 (11.17%)	85	< 0.001
PPP	75 (41.90%)	104 (58.10%)		
PPP	75 (41.90%)	104 (58.10%)	80.35	< 0.001
PPT	157 (87.71%)	22 (12.29%)		

The comparisons, between conditions, were performed using Pearson's Chi-Square test with Yates' continuity correction. P-Value is shown for all comparisons. RT – room temperature. K2EDTA - dipotassium ethylendiaminetetraacetate tubes. PPP- platelet-poor plasma. PPT - plasma-preparation tubes.

Supplementary Table 3. Detected and undetected miRNA comparison within group

STORAGE CONDITION (N = 179)	Detected miRNAs, N (%)	Undetected miRNAs, N (%)	χ² Test	P
		K2EDTA		
- 80°C	96 (53.63%)	83 (46.37%)	18.63	< 0.001
24h RT	136 (75.98%)	43 (24.02%)		
- 80°C	96 (53.63%)	83 (46.37%)	52.39	< 0.001
24h 4°C	159 (88.83%)	20 (11.17%)		
24h RT	136 (75.98%)	43 (24.02%)	9.32	0.002
24h 4°C	159 (88.83%)	20 (11.17%)		
		PPP		
- 80°C	103 (57.54%)	76 (42.46%)	9.4	0.002
24h RT	73 (40.78%)	106 (59.22%)		
- 80°C	103 (57.54%)	76 (42.46%)	8.14	0.004
24h 4°C	75 (41.90%)	104 (58.10%)		
24h RT	73 (40.78%)	106 (59.22%)	0.01	0.914
24h 4°C	75 (41.90%)	104 (58.10%)		
		PPT		
- 80°C	161 (89.94%)	18 (10.06%)	0.67	0.411
24h RT	155 (86.59%)	24 (13.41%)		
- 80°C	161 (89.94%)	18 (10.06%)	0.25	0.615
24h 4°C	157 (87.71%)	22 (12.29%)		
24h RT	155 (86.59%)	24 (13.41%)	0.02	0.874
24h 4°C	157 (87.71%)	22 (12.29%)		

The comparisons, between conditions, were performed using Pearson's Chi-Square test with Yates' continuity correction. P-Value is shown for all comparisons. RT – room temperature. K2EDTA - dipotassium ethylendiaminetetraacetate tubes. PPP- platelet-poor plasma. PPT - plasma-preparation tubes.

Supplementary Table 4. Matched detected and undetected miRNAs in between-group comparison

		- 80°C				24h RT				24h 4°C			
**N=179**		**Detected miRNAs**	**Undetected miRNAs**	**χ² Test**	**P**	**Detected miRNAs**	**Undetected miRNAs**	**χ² Test**	**P**	**Detected miRNAs**	**Undetected miRNAs**	**χ² Test**	**P**
						**K2EDTA**							
PPP	**Detected miRNAs**	75 (41.89%)	30 (16.76%)	1.25	0.262	68 (37.99%)	5 (2.79%)	52.66	< 0.001	73 (40.78%)	1 (0.56%)	81.1	< 0.001
	**Undetected miRNAs**	21 (11.73%)	53 (29.61%)			68 (37.99%)	38 (21.23%)			86 (48.04%)	19 (10.61%)		
						**K2EDTA**							
PPT	**Detected miRNAs**	93 (51.95%)	68 (37.99%)	57.69	< 0.001	126 (70.39%)	30 (16.76%)	9.02	< 0.001	145 (81.00%)	12 (6.70%)	0.038	0.844
	**Undetected miRNAs**	3 (1.68%)	15 (8.38%)			10 (5.59%)	13 (7.26%)			14 (7.82%)	8 (4.47%)		
						**PPT**							
PPP	**Detected miRNAs**	98 (54.75%)	5 (2.79%)	50.7	< 0.001	72 (40.22%)	1 (0.56%)	78.11	< 0.001	73 (40.78%)	2 (1.12%)	77.29	< 0.001
	**Undetected miRNAs**	66 (36.87%)	10 (5.59%)			83 (46.37%)	23 (12.85%)			85 (47.47%)	19 (10.61%)		

Number of matched detected and undetected miRNAs are expressed as absolute and percentage values. The comparisons, between conditions, were performed using McNeman's test Chi-Square test with continuity correction. P-Value is shown for all comparisons. RT – room temperature. K2EDTA - dipotassium ethylendiaminetetraacetate tubes. PPP- platelet-poor plasma. PPT - plasma-preparation tubes.

Supplementary Table 5. Matched detected and undetected miRNA in within-group comparison

		K2EDTA				PPP				PPT			
		**Detected miRNAs**	**Undetected miRNAs**	**χ^2 Test**	**P**	**Detected miRNAs**	**Undetected miRNAs**	**χ^2 Test**	**P**	**Detected miRNAs**	**Undetected miRNAs**	**χ^2 Test**	**P**
							- 80°C						
24h RT	**Detected miRNAs**	88 (49.16%)	48 (26.82%)	27.16	< 0.001	64 (35.75%)	9 (5.03%)	17.52	< 0.001	146 (81.56%)	9 (5.08%)	1.04	0.307
	**Undetected miRNAs**	8 (4.47%)	35 (19.55%)			39 (21.79%)	67 (37.43%)			15 (8.38%)	9 (5.08%)		
							- 80°C						
24h 4°C	**Detected miRNAs**	93 (51.95%)	66 (36.87%)	55.71	< 0.001	66 (36.87%)	9 (5.03%)	15.48	< 0.001	144 (80.45%)	12 (6.70%)	0.55	0.458
	**Undetected miRNAs**	3 (1.68%)	17 (9.50%)			37 (20.67%)	67 (37.43%)			17 (9.50%)	6 (3.35%)		
							24h RT						
24h 4°C	**Detected miRNAs**	129 (72.07%)	30 (16.76%)	13.08	< 0.001	61 (34.08%)	13 (7.26%)	0	1.000	144 (80.45%)	13 (7.26%)	0.04	0.838
	**Undetected miRNAs**	7 (3.91%)	13 (7.26%)			12 (6.70%)	93 (51.95%)			11 (6.14%)	11 (6.14%)		

Number of detected and undetected miRNAs are expressed as absolute and percentage values. The comparisons, between conditions, were performed using McNemar' s Chi-Square test with continuity correction. P-Value is shown for all comparisons. RT – room temperature. K2EDTA - dipotassium ethylendiaminetetraacetate tubes. PPP- platelet-poor plasma. PPT - plasma-preparation tubes.

Supplementary Table 6. Within-group comparison of circulating miRNA expression

	WITHIN-GROUP ANALYSIS											
miRNAs,	K2EDTA				PPP				PPT			
fold change (P)	24h RT *vs* - 80°C	24h 4°C *vs* - 80°C	24h 4°C *vs* 24h RT	P	24h RT *vs* - 80°C	24h 4°C *vs* - 80°C	24h 4°C *vs* 24h RT	P	24h RT *vs* - 80°C	24h 4°C *vs* - 80°C	24h 4°C *vs* 24h RT	P
**hsa-let-7a-5p**	2.08 (0.051)	1.11 (0.990)	0.53 (0.303)	0.051	0.87	0.82	0.93	0.67	1.21 (0.990)	0.54 (0.303)	0.45 (0.051)	0.051
**hsa-let-7b-3p**	1.29	1.17	0.91	0.782	1.00	1.00	1.00	1.000	0.48 (0.990)	0.04 (0.051)	0.08 (0.303)	0.051
**hsa-let-7b-5p**	1.56	1.10	0.71	0.113	0.26 (0.022)	0.49 (0.539)	1.89 (0.539)	0.027	0.55	0.55	0.98	0.067
**hsa-let-7c-5p**	2.00	0.77	0.38	0.113	0.02	0.02	0.97	0.064	0.92	0.40	0.44	0.252
**hsa-let-7d-3p**	0.64	0.59	0.92	0.118	1.29	1.61	1.25	0.288	1.68	1.31	0.78	0.252
**hsa-let-7d-5p**	2.64	1.49	0.56	0.148	0.02	0.76	36.75	0.061	0.92	0.72	0.78	0.491
**hsa-let-7e-5p**	62.89	72.78	1.16	0.067	0.01	0.95	78.05	0.067	1.41	0.72	0.51	0.252
**hsa-let-7f-5p**	1.67	0.77	0.46	0.252	1.19	0.83	0.70	0.252	1.32	0.72	0.54	0.113
**hsa-let-7g-5p**	1.15	1.11	0.96	0.875	0.86	0.59	0.69	0.252	0.76 (0.990)	0.43 (0.051)	0.56 (0.303)	0.051
**hsa-let-7i-5p**	1.61	0.93	0.57	0.43	1.05	1.32	1.25	0.491	1.02	0.72	0.70	0.43
**hsa-miR-1**	51.98 (0.303)	57.33 (0.051)	1.10 (0.990)	0.051	0.02	0.02	1.00	0.106	0.48	0.90	1.86	0.55
**hsa-miR-101-3p**	0.18 (0.051)	0.39 (0.408)	2.13 (0.890)	0.051	1.08	1.12	1.04	0.733	0.53	0.76	1.44	0.561
**hsa-miR-103a-3p**	0.56	0.46	0.81	0.061	2.20	1.13	0.51	0.113	1.55	0.69	0.45	0.099
**hsa-miR-106a-5p**	0.57 (0.408)	1.18 (0.890)	2.07 (0.034)	0.039	0.61	1.53	2.52	0.113	1.51	0.51	0.33	0.067
**hsa-miR-106b-3p**	1.34	58.43	43.52	0.058	0.74	0.74	1.00	0.723	1.46 (0.890)	0.04 (0.408)	**0.03 (0.034)**	0.039
**hsa-miR-106b-5p**	0.47	0.77	1.64	0.587	0.00	0.77	319.28	0.067	0.45	0.85	1.89	0.329
**hsa-miR-107**	0.80	0.44	0.55	0.329	1.04	0.58	0.55	0.113	0.73	0.83	1.14	0.837

**hsa-miR-10b-5p**	0.78	0.92	1.17	0.782	**253.03 (0.022)**	186.35 (0.539)	0.74 (0.539)	0.027	18.71 (0.303)	32.18 (0.051)	1.72 (0.539)	0.051
**hsa-miR-122-5p**	0.36	0.83	2.32	0.113	0.45	0.69	1.55	0.079	0.15	0.34	2.24	0.058
**hsa-miR-125a-5p**	1.25	0.47	0.37	0.099	**123.58 (0.022)**	58.10 (0.539)	0.47 (0.539)	0.027	1.66	1.29	0.77	0.148
**hsa-miR-125b-5p**	**0.005 (0.022)**	0.21 (0.539)	40.46 (0.539)	0.027	0.89	0.89	1.00	0.965	0.51 (0.539)	**0.03 (0.022)**	0.05 (0.539)	0.027
**hsa-miR-1260a**	92.77	67.38	0.73	0.061	0.94	0.94	1.00	0.723	0.91	1.23	1.36	0.393
**hsa-miR-126-3p**	0.79	0.72	0.92	0.193	1.40	1.07	0.76	0.177	0.91 (0.539)	0.74 (0.022)	0.81 (0.539)	0.027
**hsa-miR-126-5p**	1.62	1.17	0.72	0.393	0.91	0.46	0.50	0.43	2.01	2.16	1.07	0.067
**hsa-miR-127-3p**	40.96	40.81	1.00	0.088	1.00	1.00	1.00	1.000	2.02	1.17	0.58	0.061
**hsa-miR-128-3p**	1.00	51.59	51.59	0.063	1.13	1.28	1.13	0.243	53.70	36.58	0.68	0.061
**hsa-miR-130a-3p**	56.18	54.99	0.98	0.067	1.81	1.60	0.88	0.634	1.94	2.04	1.05	0.288
**hsa-miR-130b-3p**	0.60	14.36	23.84	0.074	0.76	0.76	1.00	0.723	1.00	1.00	1.00	1.000
**hsa-miR-132-3p**	144.39 (0.051)	53.38 (0.303)	0.37 (0.990)	0.051	0.92	0.97	1.05	0.782	1.83 (0.408)	2.69 (0.034)	1.47 (0.890)	0.039
**hsa-miR-133b**	1.00	1.75	1.75	0.106	1.00	1.00	1.00	1.000	1.00	26.98	26.98	0.063
**hsa-miR-139-5p**	0.96	0.84	0.87	0.957	1.00	1.05	1.05	0.869	0.93	0.56	0.60	0.099
**hsa-miR-140-3p**	0.61	1.91	3.11	0.099	0.82	1.74	2.12	0.113	0.46	1.10	2.37	0.067
**hsa-miR-140-5p**	1.06	1.00	0.94	0.723	1.06	1.10	1.04	0.243	0.85	1.00	1.18	0.301
**hsa-miR-142-3p**	**0.14 (0.034)**	0.31 (0.408)	2.13 (0.890)	0.039	0.55 (0.990)	0.24 (0.051)	0.44 (0.303)	0.051	0.33 (0.051)	0.77 (0.990)	2.34 (0.303)	0.051
**hsa-miR-142-5p**	0.50	0.36	0.72	0.061	1.00	2.09	2.09	0.723	0.87	1.27	1.46	0.837
**hsa-miR-143-3p**	1.00	74.97	74.97	0.063	0.01 (0.470)	**0.01 (0.027)**	0.65 (0.699)	0.032	0.84	2.15	2.57	0.177
**hsa-miR-144-3p**	0.38	0.59	1.56	0.113	4.15	2.37	0.57	0.393	1.14	5.71	5.00	0.118
**hsa-miR-144-5p**	105.23	101.23	0.96	0.061	1.04	170.82	164.14	0.064	0.87	0.51	0.58	0.301
**hsa-miR-145-5p**	1.31	0.76	0.58	0.561	2.43 (0.022)	1.63 (0.539)	0.67 (0.539)	0.027	1.67	1.16	0.70	0.301
**hsa-miR-146a-5p**	1.29	1.18	0.92	0.67	2.73 (0.051)	2.18 (0.303)	0.80 (0.990)	0.051	2.69	1.78	0.66	0.148

**hsa-miR-146b-5p**	98.96	77.99	0.79	0.061	0.01	0.01	1.03	0.064	1.46	1.97	1.35	0.43
**hsa-miR-148a-3p**	113.65	118.54	1.04	0.067	1.00	1.00	1.00	1.000	1.01	1.07	1.06	0.67
**hsa-miR-148b-3p**	1.00	158.27	158.27	0.063	1.61 (0.539)	0.01 (0.539)	**0.003 (0.022)**	0.027	1.54	1.29	0.84	0.875
**hsa-miR-150-5p**	1.07	0.88	0.83	0.875	1.19	1.34	1.13	0.288	0.71	1.34	1.88	0.43
**hsa-miR-151a-3p**	1.65	1.41	0.86	0.288	1.33	0.03	0.02	0.061	1.16	0.96	0.83	0.957
**hsa-miR-151a-5p**	0.71	0.52	0.73	0.491	1.42	0.76	0.53	0.202	1.55	1.17	0.76	0.393
**hsa-miR-152-3p**	0.93	0.30	0.32	0.061	178.87	0.59	0.00	0.058	2.03	1.71	0.84	0.148
**hsa-miR-154-5p**	1.43	1.01	0.71	0.243	1.00	1.00	1.00	1.000	0.94	0.03	0.03	0.061
**hsa-miR-155-5p**	85.98	0.91	0.01	0.064	0.01	0.01	1.10	0.064	0.92	1.08	1.17	0.875
**hsa-miR-15a-5p**	2.55	2.53	0.99	0.067	1.20	0.60	0.50	0.099	1.05	0.56	0.53	0.118
**hsa-miR-15b-3p**	58.60 (0.408)	**85.52 (0.034)**	1.46 (0.990)	0.039	1.01	150.74	149.81	0.064	0.50	0.72	1.43	0.079
**hsa-miR-15b-5p**	0.86	0.79	0.92	0.731	0.02	0.01	0.71	0.058	1.52	1.44	0.95	0.733
**hsa-miR-16-2-3p**	80.32	75.56	0.94	0.067	0.01	0.01	0.88	0.058	0.02	0.02	1.00	0.063
**hsa-miR-16-5p**	0.90 (0.539)	1.10 (0.539)	1.22 (0.022)	0.027	1.23 (0.051)	1.74 (0.990)	1.41 (0.303)	0.051	0.84 (0.890)	1.80 (0.408)	2.15 (0.034)	0.039
**hsa-miR-17-5p**	1.02	62.42	61.43	0.064	1.00	1.00	1.00	1.000	0.86 (0.990)	2.28 (0.303)	2.65 (0.051)	0.051
**hsa-miR-181a-5p**	0.90 (0.990)	0.61 (0.303)	0.68 (0.051)	0.491	0.02	0.03	1.09	0.064	0.76	0.33	0.44	0.252
**hsa-miR-185-5p**	0.61	0.41	0.67	0.051	0.22	0.79	3.51	0.061	1.17 (0.990)	2.19 (0.051)	1.87 (0.303)	0.051
**hsa-miR-186-5p**	59.17	1.00	0.02	0.063	0.47	0.47	1.00	0.57	17.33	1.00	0.06	0.063
**hsa-miR-18a-5p**	1.03	0.81	0.79	0.587	0.03	0.02	0.83	0.063	1.21	1.22	1.01	0.837
**hsa-miR-18b-5p**	0.60	0.55	0.92	0.118	1.44	1.00	0.69	0.723	1.12	1.01	0.91	0.957
**hsa-miR-191-5p**	2.45	1.20	0.49	0.061	1.41	1.44	1.02	0.252	1.45	1.03	0.71	0.113
**hsa-miR-192-5p**	0.43	0.90	2.10	0.067	0.75	0.72	0.95	0.193	0.63	0.70	1.11	0.301
**hsa-miR-193a-5p**	0.96	1.28	1.34	0.634	0.59	0.78	1.32	0.243	0.04	0.04	0.23	0.063
**hsa-miR-194-5p**	54.56	57.58	1.06	0.061	1.71	0.82	0.48	0.099	0.73	1.05	1.44	0.561
**hsa-miR-195-5p**	66.86	57.69	0.86	0.061	1.00	0.55	0.55	0.723	0.78	3.07	3.94	0.252

**hsa-miR-197-3p**	1.28	1.38	1.07	0.561	3.06 (0.051)	2.22 (0.303)	0.73 (0.990)	0.051	1.96	1.62	0.83	0.733
**hsa-miR-199a-3p**	1.83	1.21	0.66	0.733	1.00	61.46	61.46	0.063	2.06	0.70	0.34	0.177
**hsa-miR-199a-5p**	0.02	0.02	1.00	0.063	0.87	0.87	1.00	0.965	1.74	1.77	1.02	0.561
**hsa-miR-19a-3p**	**0.16 (0.022)**	0.39 (0.539)	2.43 (0.539)	0.027	1.04	2.37	2.27	0.393	0.95	4.36	4.59	0.061
**hsa-miR-19b-3p**	0.12	0.88	7.30	0.061	0.67	1.17	1.74	0.733	0.60	1.16	1.93	0.43
**hsa-miR-200c-3p**	88.60	38.04	0.43	0.094	0.78	0.78	1.00	0.723	1.28	1.83	1.43	0.957
**hsa-miR-205-5p**	1.00	41.41	41.41	0.063	0.01	1.10	99.72	0.105	0.83	0.80	0.96	0.832
**hsa-miR-20a-5p**	**0.30 (0.022)**	0.49 (0.539)	1.65 (0.539)	0.027	0.30	0.93	3.10	0.061	1.47	1.97	1.33	0.202
**hsa-miR-210-3p**	1.38	74.87	54.15	0.058	0.01	0.01	1.00	0.063	0.05	0.05	1.00	0.063
**hsa-miR-2110**	97.42	95.16	0.98	0.067	0.01	0.01	1.05	0.107	0.92	1.06	1.16	0.957
**hsa-miR-215-5p**	**0.005 (0.022)**	0.38 (0.539)	80.98 (0.539)	0.027	0.00	1.08	227.26	0.061	0.44	0.69	1.59	0.329
**hsa-miR-21-5p**	0.93	1.81	1.95	0.061	1.20	0.38	0.32	0.061	0.67 (0.539)	1.84 (0.539)	2.73 (0.022)	0.027
**hsa-miR-221-3p**	0.44	0.41	0.93	0.288	1.83	2.06	1.13	0.061	2.47	1.41	0.57	0.252
**hsa-miR-222-3p**	0.98	1.37	1.39	0.837	2.31	1.31	0.57	0.202	0.97	1.58	1.64	0.837
**hsa-miR-223-3p**	1.65 (0.022)	2.44 (0.539)	1.47 (0.539)	0.027	**0.18 (0.022)**	0.59 (0.539)	3.25 (0.539)	0.027	1.68	1.52	0.91	0.059
**hsa-miR-223-5p**	1.00	62.65	62.65	0.063	1.00	1.00	1.00	1.000	1.10	0.03	0.03	0.067
**hsa-miR-22-3p**	0.56	0.68	1.21	0.561	1.39	1.15	0.83	0.329	1.34	1.58	1.18	0.733
**hsa-miR-23a-3p**	1.78	1.95	1.09	0.067	1.06	1.40	1.32	0.067	1.91 (0.022)	1.56 (0.539)	0.81 (0.539)	0.027
**hsa-miR-23b-3p**	2.05	2.17	1.06	0.067	1.62	0.68	0.42	0.148	1.17	1.60	1.36	0.099
**hsa-miR-24-3p**	1.62	0.97	0.60	0.061	1.15	2.23	1.94	0.061	1.19 (0.990)	1.54 (0.051)	1.30 (0.303)	0.051
**hsa-miR-25-3p**	0.80	0.86	1.08	0.148	0.84	0.98	1.17	0.587	0.56 (0.034)	0.91 (0.890)	1.63 (0.408)	0.039
**hsa-miR-26a-5p**	1.16	0.77	0.66	0.561	1.17 (0.990)	0.67 (0.303)	0.58 (0.051)	0.051	1.39	1.85	1.33	0.079

**hsa-miR-26b-5p**	0.60	0.29	0.49	0.177	0.75	0.01	0.01	0.058	1.05	0.93	0.88	0.733
**hsa-miR-27a-3p**	0.86	1.12	1.31	0.393	0.02	0.02	1.07	0.064	1.17	1.71	1.46	0.252
**hsa-miR-27b-3p**	0.67	0.74	1.10	0.252	1.10	1.12	1.02	0.957	1.69	1.64	0.97	0.193
**hsa-miR-28-3p**	45.29	36.91	0.81	0.061	1.00	0.73	0.73	0.723	0.92	0.87	0.94	0.733
**hsa-miR-28-5p**	1.38	0.82	0.89	0.393	1.00	1.00	1.00	1.000	1.12	0.86	0.77	0.561
**hsa-miR-29a-3p**	0.42	0.63	1.50	0.113	2.09	0.86	0.41	0.061	0.84	0.99	1.18	0.733
**hsa-miR-29b-3p**	1.26 (0.699)	**90.01 (0.027)**	71.33 (0.470)	0.032	1.13	1.13	1.00	0.723	0.03	0.03	1.00	0.063
**hsa-miR-29c-3p**	105.48	155.82	1.48	0.061	0.00	0.00	1.00	0.063	0.02 (0.408)	2.10 (0.890)	**116.47 (0.034)**	0.039
**hsa-miR-301a-3p**	0.41 (0.408)	0.26 (0.034)	0.63 (0.890)	0.039	0.98	0.98	1.00	0.965	0.82	1.83	2.24	0.113
**hsa-miR-30a-5p**	0.94	43.97	46.60	0.064	**361.27 (0.022)**	213.25 (0.539)	0.59 (0.539)	0.027	1.11	1.46	1.31	0.733
**hsa-miR-30b-5p**	1.26	1.14	0.90	0.875	1.27	1.48	1.17	0.587	1.32	0.82	0.62	0.148
**hsa-miR-30c-5p**	1.24	0.56	0.45	0.099	1.33	1.28	0.96	0.733	0.86	0.56	0.65	0.079
**hsa-miR-30d-5p**	1.62	1.22	0.75	0.252	1.40	1.41	1.01	0.301	1.72	1.37	0.80	0.113
**hsa-miR-30e-3p**	94.44	56.99	0.60	0.061	0.01	0.01	0.95	0.064	1.13	2.61	2.31	0.43
**hsa-miR-30e-5p**	1.08	1.09	1.01	0.957	1.13	1.29	1.14	0.67	1.52	1.28	0.84	0.957
**hsa-miR-320a**	1.96 (0.022)	1.20 (0.539)	0.61 (0.539)	0.027	2.23 (0.303)	2.46 (0.051)	1.10 (0.990)	0.051	0.92	0.76 (0.034)	0.83	0.039
**hsa-miR-320b**	3.36 (0.034)	1.38	0.41	0.039	1.08	1.73	1.59	0.061	1.31	0.93	0.71	0.202
**hsa-miR-320c**	2.03	1.10	0.54	0.067	1.45	1.01	0.70	0.301	1.34	1.11	0.83	0.875
**hsa-miR-320d**	1.35	0.58	0.43	0.061	2.35	1.05	0.45	0.061	1.40	1.43	1.02	0.875
**hsa-miR-324-3p**	**82.55 (0.022)**	45.45 (0.539)	0.55 (0.539)	0.027	1.02	1.02	1.00	0.965	0.02	0.02	1.00	0.063
**hsa-miR-324-5p**	1.00	1.00	1.00	0.999	1.02	1.02	1.00	0.965	0.82	0.77	0.94	0.733
**hsa-miR-32-5p**	0.84 (0.539)	**0.02 (0.022)**	0.02 (0.539)	0.027	0.01	0.01	1.10	0.064	**25.95 (0.042)**	6.99 (0.539)	0.27 (0.539)	0.047
**hsa-miR-326**	52.65	35.50	0.67	0.105	0.01	0.01	1.00	0.106	1.12	0.87	0.78	0.925
**hsa-miR-328-3p**	1.31	0.91	0.69	0.957	1.06	1.00	0.95	0.965	0.97	0.99	1.02	0.957

**hsa-miR-331-3p**	0.51 (0.539)	0.28 (0.022)	0.54 (0.539)	0.027	0.01	0.01	1.00	0.063	68.65	63.38	0.92	0.067
**hsa-miR-335-3p**	**109.94 (0.022)**	40.61 (0.539)	0.37 (0.539)	0.027	0.86	0.86	1.00	0.723	0.70	1.10	1.56	0.329
**hsa-miR-338-3p**	0.01	0.26	21.32	0.092	1.00	1.00	1.00	1.000	4.85	2.94	0.61	0.172
**hsa-miR-339-3p**	1.22	1.13	0.92	0.243	1.00	1.00	1.00	1.000	0.81	0.85	1.06	0.733
**hsa-miR-339-5p**	0.83	0.46	0.55	0.099	1.00	1.20	1.20	0.723	1.44	0.86	0.60	0.875
**hsa-miR-342-3p**	0.47	0.53	1.11	0.113	0.78	1.06	1.36	0.099	0.89	0.63	0.70	0.113
**hsa-miR-34a-5p**	0.01	0.01	1.00	0.106	0.02	0.02	0.02	0.106	0.46	2.51	5.48	0.088
**hsa-miR-361-5p**	41.21	40.19	0.98	0.067	0.018 (0.051)	0.71 (0.990)	38.93 (0.303)	0.051	1.49	1.34	0.90	0.202
**hsa-miR-363-3p**	0.63	1.48	2.36	0.252	426.24 (0.051)	293.57 (0.303)	0.69 (0.990)	0.051	0.97	2.06	2.12	0.301
**hsa-miR-374a-5p**	62.74	58.00	0.92	0.067	1.16	1.16	1.00	0.723	0.99	1.75	1.76	0.43
**hsa-miR-374b-5p**	0.76	1.08	1.42	0.067	1.08	1.08	1.00	0.965	1.29	0.85	0.66	0.288
**hsa-miR-375**	0.87	0.25	0.29	0.061	0.95	537.81	567.13	0.064	0.85 (0.990)	0.12 (0.051)	0.14 (0.303)	0.051
**hsa-miR-376a-3p**	**119.83 (0.022)**	60.29 (0.539)	0.50 (0.539)	0.027	1.10	1.06	0.96	0.243	1.45	1.12	0.77	0.733
**hsa-miR-376c-3p**	48.18	37.90	0.79	0.067	1.00	1.00	1.00	1.000	1.20	1.31	1.09	0.587
**hsa-miR-378a-3p**	0.43	0.53	1.24	0.118	1.03	0.59	0.58	0.249	1.16	2.13	1.84	0.113
**hsa-miR-382-5p**	**48.84 (0.034)**	34.56 (0.408)	0.71 (0.890)	0.039	1.00	1.00	1.00	1.000	1.30	1.19	0.91	0.733
**hsa-miR-409-3p**	167.47	54.75	0.33	0.061	0.93	0.93	1.00	0.723	1.08	1.15	1.07	0.957
**hsa-miR-421**	77.44	59.37	0.77	0.061	1.00	1.00	1.00	1.000	1.18	1.20	1.01	0.875
**hsa-miR-423-3p**	255.60	312.52	1.22	0.067	1.00	1.23	1.23	0.723	1.17	1.19	1.01	0.733
**hsa-miR-423-5p**	1.79	1.71	0.95	0.393	1.63	1.52	0.93	0.43	1.26	0.80	0.63	0.43
**hsa-miR-424-5p**	**0.01 (0.022)**	0.42 (0.539)	53.00 (0.539)	0.027	291.39	1.00	0.00	0.063	26.76	1.00	0.04	0.063
**hsa-miR-425-3p**	**70.16 (0.022)**	36.94 (0.539)	0.53 (0.539)	0.027	0.93	0.93	1.00	0.723	1.46	0.95	0.65	0.193
**hsa-miR-425-5p**	157.54	102.89	0.65	0.061	0.01	0.71	132.01	0.061	1.05	1.00	0.94	0.957

**hsa-miR-451a**	0.18 (0.051)	0.57 (0.990)	3.19 (0.303)	0.051	0.53	0.55	1.04	0.067	0.38	0.70	1.83	0.202
**hsa-miR-454-3p**	**85.10 (0.034)**	48.15 (0.408)	0.57 (0.890)	0.039	0.91	0.91	1.00	0.723	0.85	0.61	0.72	0.252
**hsa-miR-483-5p**	120.24	51.37	0.43	0.061	0.99	0.00	0.00	0.061	0.82	1.26	1.53	0.561
**hsa-miR-484**	0.67	0.61	0.91	0.193	1.61 (0.034)	1.20	0.74	0.039	1.46	1.30	0.89	0.733
**hsa-miR-485-3p**	**102.79 (0.034)**	52.17 (0.408)	0.51 (0.890)	0.039	1.00	1.00	1.00	1.000	0.88	0.01	0.02	0.061
**hsa-miR-486-5p**	2.55	1.73	0.68 (0.051)	0.051	1.07	1.51	1.41	0.061	0.34 (0.022)	0.88 (0.539)	2.56 (0.539)	0.027
**hsa-miR-495-3p**	29.29	17.06	0.58	0.292	0.04	0.04	1.00	0.106	0.89	2.95	3.30	0.55
**hsa-miR-501-3p**	94.77	65.85	0.69	0.061	0.78	0.78	1.00	0.723	0.93	20.77	22.43	0.063
**hsa-miR-502-3p**	40.99	1.31	0.03	0.058	1.00	1.00	1.00	1.000	1.00	15.02	15.02	0.063
**hsa-miR-505-3p**	173.71	95.00	0.55	0.061	0.89	0.89	1.00	0.965	0.94	0.03	0.03	0.067
**hsa-miR-532-3p**	0.85 (0.022)	66.76 (0.539)	78.56 (0.539)	0.058	0.76	0.92	1.21	0.243	1.56	0.03	0.02	0.061
**hsa-miR-532-5p**	0.02	0.52	29.04	0.027	0.01 (0.303)	1.10 (0.990)	109.65 (0.051)	0.051	0.56	1.20	2.14	0.301
**hsa-miR-543**	31.81	19.19		0.105	1.00	1.00	1.00	1.000	5.90	1.68	0.29	0.063
**hsa-miR-574-3p**	0.30 (0.034)	0.54 (0.408)	1.81 (0.890)	0.039	0.02 (0.470)	**0.01 (0.027)**	0.80 (0.699)	0.032	0.84	0.78	0.93	0.67
**hsa-miR-584-5p**	75.16	43.66	0.58	0.061	0.91	0.91	1.00	0.723	1.46	1.27	0.87	0.252
**hsa-miR-590-5p**	0.41 (0.051)	0.75 (0.990)	1.83 (0.303)	0.051	1.27	1.05	0.83	0.193	1.06	1.60	1.50	0.43
**hsa-miR-629-5p**	0.81	0.89	1.09	0.561	2.91 (0.470)	0.01 (0.699)	**0.004 (0.027)**	0.032	0.91	1.98	2.18	0.707
**hsa-miR-652-3p**	1.55	0.62	0.40	0.177	1.18	1.33	1.13	0.179	1.01	0.71	0.70	0.43
**hsa-miR-660-5p**	47.80	55.51	1.16	0.067	164.86	0.92	0.01	0.055	1.00	30.22	30.22	0.063
**hsa-miR-7-5p**	1.00	41.95		0.063	0.02	0.02	1.00	0.106	0.48	0.47	0.99	0.55
**hsa-miR-766-3p**	1.24	0.67	0.54	0.202	1.00	1.00	1.00	0.965	1.87	4.33	2.31	0.061
**hsa-miR-874-3p**	51.27	1.32	0.03	0.058	0.76	0.76	1.00	0.723	0.06	0.08	1.32	0.058

**hsa-miR-877-5p**	0.72	86.06	119.53	0.055	0.01	0.01	1.34	0.063	0.64	1.29	2.01	0.43
**hsa-miR-885-5p**	1.00	45.46	45.46	0.063	0.01	0.01	1.00	0.063	0.86	3.72	4.34	0.707
**hsa-miR-92a-3p**	1.83 (0.539)	1.94 (0.022)	1.06 (0.539)	0.027	2.25 (0.022)	1.74 (0.539)	0.77 (0.539)	0.027	1.11	0.91	0.82	0.288
**hsa-miR-92b-3p**	105.57	1.00	0.01	0.063	0.77	0.77	1.00	0.723	0.04	1.22	30.53	0.067
**hsa-miR-93-3p**	70.27	77.79	1.11	0.061	0.01 (0.539)	1.54 (0.539)	**156.47 (0.022)**	0.027	0.46	0.76	1.64	0.561
**hsa-miR-93-5p**	0.57	0.59	1.03	0.061	0.85	1.43	1.69	0.202	0.72	1.71	2.37	0.099
**hsa-miR-99a-5p**	**93.07 (0.034)**	48.88 (0.408)	0.53 (0.890)	0.039	0.87	1.00	1.15	0.723	38.95	33.56	0.86	0.061
**hsa-miR-99b-5p**	1.88	0.80	0.42	0.113	1.06	1.06	1.00	0.106	1.44	0.88	0.61	0.587

Expressions for each miRNAs from K2EDTA, PPP and PPT samples and each storage condition were compared through Kruskal-Wallis test. $P < 0.05$ was considered statistically significant. Dunn's multiple comparison test was performed for the comparison between two conditions, only for significant Kruskal-Wallis test. Differences in miRNA expression between two conditions is reported as the ratio of miRNA expression of an "experimental" condition and a "reference" condition (fold change). In bold are reported ≥ ± 5-fold change values which are statistically significant. RT – room temperature. K2EDTA - dipotassium ethylendiaminetetraacetate tubes. PPT – plasma preparation tubes. PPP- platelet-poor plasma.
